# Protein analysis reveals differential accumulation of late embryogenesis abundant and storage proteins in seeds of wild and cultivated amaranth species

**DOI:** 10.1186/s12870-019-1656-7

**Published:** 2019-02-06

**Authors:** Esaú Bojórquez-Velázquez, Alberto Barrera-Pacheco, Eduardo Espitia-Rangel, Alfredo Herrera-Estrella, Ana Paulina Barba de la Rosa

**Affiliations:** 1 Instituto Potosino de Investigación Científica y Tecnológica, A.C, 78216 San Luis Potosí, Mexico; 2 Instituto Nacional de Investigaciones Forestales Agrícolas y Pecuarias, 56250 Texcoco, Estado de México Mexico; 3 Laboratorio Nacional de Genómica para la Biodiversidad, CINVESTAV-Irapuato, 36821 Guanajuato, Mexico

**Keywords:** Amaranth species, Late embryogenesis abundant proteins, Proteomics, Seed storage proteins, 11S globulins

## Abstract

**Background:**

Amaranth is a plant naturally resistant to various types of stresses that produces seeds of excellent nutritional quality, so amaranth is a promising system for food production. Amaranth wild relatives have survived climate changes and grow under harsh conditions, however no studies about morphological and molecular characteristics of their seeds are known. Therefore, we carried out a detailed morphological and molecular characterization of wild species *A. powellii* and *A. hybridus*, and compared them with the cultivated amaranth species *A. hypochondriacus* (waxy and non-waxy seeds) and *A. cruentus.*

**Results:**

Seed proteins were fractionated according to their polarity properties and were analysed in one-dimensional gel electrophoresis (1-DE) followed by nano-liquid chromatography coupled to tandem mass spectrometry (nLC-MS/MS). A total of 34 differentially accumulated protein bands were detected and 105 proteins were successfully identified. Late embryogenesis abundant proteins were detected as species-specific. Oleosins and oil bodies associated proteins were observed preferentially in *A. cruentus*. Different isoforms of the granule-bound starch synthase I, and several paralogs of 7S and 11S globulins were also identified. The in silico structural analysis from different isoforms of 11S globulins was carried out, including new types of 11S globulin not reported so far.

**Conclusions:**

The results provide novel information about 11S globulins and proteins related in seed protection, which could play important roles in the nutritional value and adaptive tolerance to stress in amaranth species.

**Electronic supplementary material:**

The online version of this article (10.1186/s12870-019-1656-7) contains supplementary material, which is available to authorized users.

## Background

Food security is threatened by both the growing human population, estimated to reach around 9.3 billion by the year 2050, and the loss of crops due to climate changes and soil deterioration [1, 2]. Seeds are the centre to crop production, human nutrition, and food security [3, 4], they contain the full genetic complement of the plant allowing it to survive even under prolonged periods of stress conditions [5, 6]. Then it is of important concern to collect and preserve the germplasm of commercial species as well as their wild relatives, which have survived several climate changes and are valuable resources of genetic information that could be useful in the development of crop breeding strategies to solve current and future agricultural challenges [1, 3, 4].

Orthodox seeds are able to survive the removal of most of their cellular water and can be stored in dry state for a long period of time. Desiccation tolerance and maintenance of seeds quiescent state are associated with wide range of systems related with cell protection, detoxification, and repair [6, 7]. The presence of particular proteins such as the late embryogenesis abundant (LEA) proteins, heat shock proteins (HSPs), and seed storage proteins (SSPs) confer seeds desiccation tolerance, allowing them to survive in dry state preserving their germination ability and propagation after long-term storage conditions [8, 9].

LEA proteins are suggested to play an important role in seed desiccation tolerance [10], they are known to stabilize membranes against the deleterious effects of drying. Further, LEAs are able to prevent protein aggregation during freezing and drying and interact with and stabilize liposomes in the dry state [11]. Some LEAs can stabilize sugar glasses [12] suggesting that they play a role in longevity, which is a crucial factor for the conservation of genetic resources and to ensure proper seedling establishment and crop yield [13]. On the other hand, SSPs are a major source of dietary protein for human nutrition. SSPs beyond serving as a nutrient reservoir they may play specific functions during seed formation [7, 14] and could have a key role in seed longevity [15]. SSPs play a fundamental role in germination and seedling growth [16]. Due to their abundance and high propensity to oxidation, SSPs are considered a powerful reactive oxygen species (ROS) scavenging system that could protect cellular components that are important for embryo survival [17, 18].

Amaranth is a crop that had great importance for Aztec, Mayan, and Inca cultures. However, Spaniards prohibited its cultivation due to its link with pagan ceremonies [19]. Nevertheless, during the past two decades, reports on amaranth nutritional and nutraceutical characteristics have increased, leading to a new era in the history of amaranth cultivation [20]. The importance of amaranth as a crop for human nutrition is due to the high quality of its proteins. Amaranth seed proteins contain an adequate balance of essential amino acids [21], with values close to nutritional human requirements, being particularly rich in lysine and methionine, which are deficient in cereals and legumes, respectively [20, 22]. Furthermore, the content of prolamins, the SSPs fraction responsibles for the manifestation of celiac disease, is negligible or practically null [23]. The genus *Amaranthus* consists of about 70 species distributed in very diverse habitats in terms of climatic conditions and geographical location [24, 25], of which only three species, *A. caudatus*, *A. cruentus,* and *A. hypochondriacus* are cultivated as grain amaranths for human consumption, the last two being native to Mexico [26]. The most probable ancestors or wild relatives of these species are *A. powellii* and *A. hybridus*, which grow under harsh conditions throughout the Mexican territory. The wide natural variation in amaranth offers the opportunity to identify markers that could be important for the nutrition, protection and longevity of seeds, which would result in the development of high productivity cultivars.

The aim of this study was to characterize the morphological and molecular traits of seeds from wild species *A. powellii* and *A. hybridus* and compared them with the cultivated amaranth species such as *A. hypochondriacus* and *A. cruentus.* The seeds phenotypic analysis was carried by microscopy observations and molecular characterization was carried out using proteomics tools (1-DE and nLC-MS/MS) as well as in silico analyses.

## Results

### Amaranth wild species present non-waxy phenotype

**Fig. 1:**
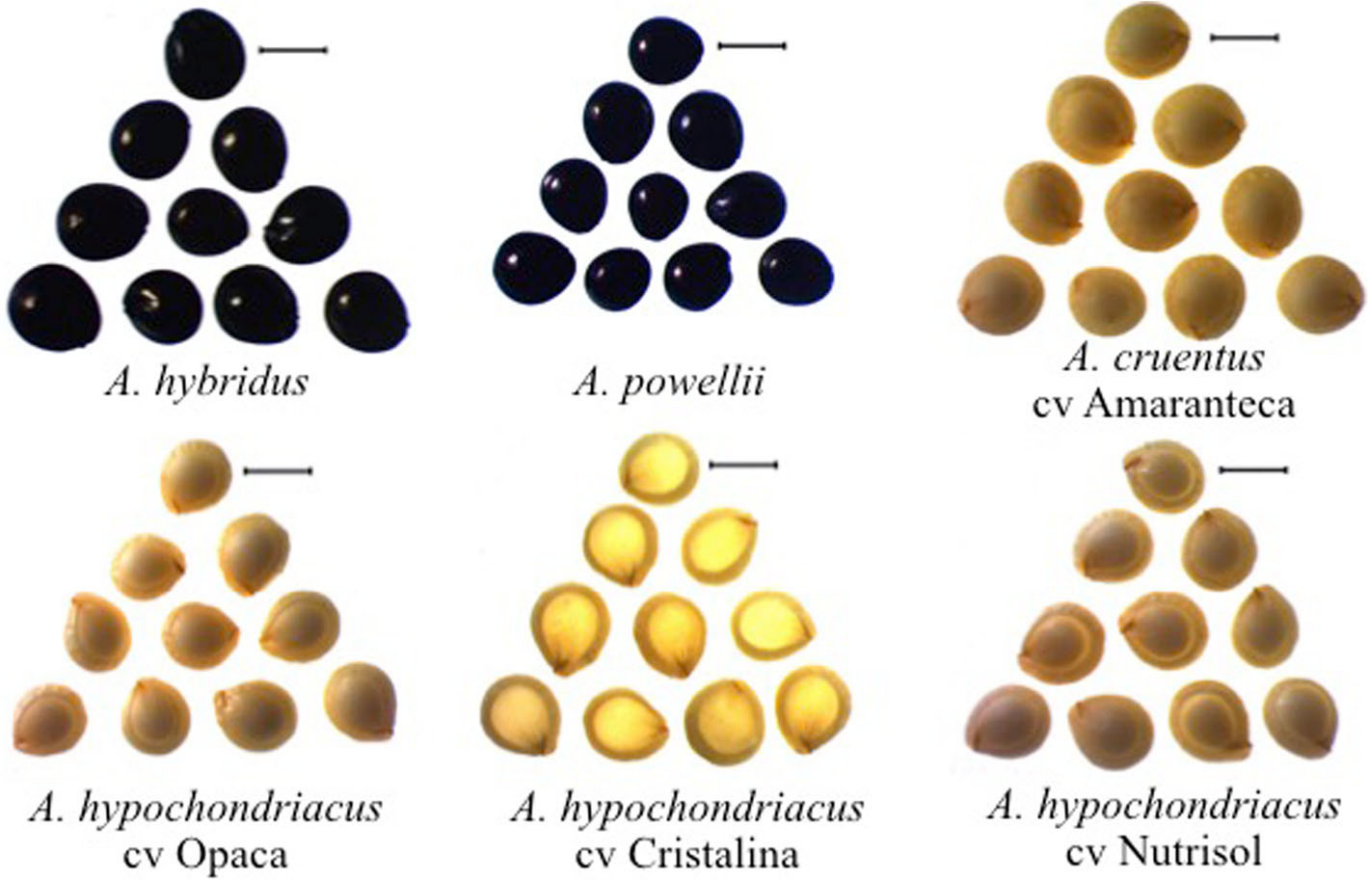
Morphological characteristics of intact seeds from wild and cultivated amaranth species. Bars 1 mm

**Fig. 2:**
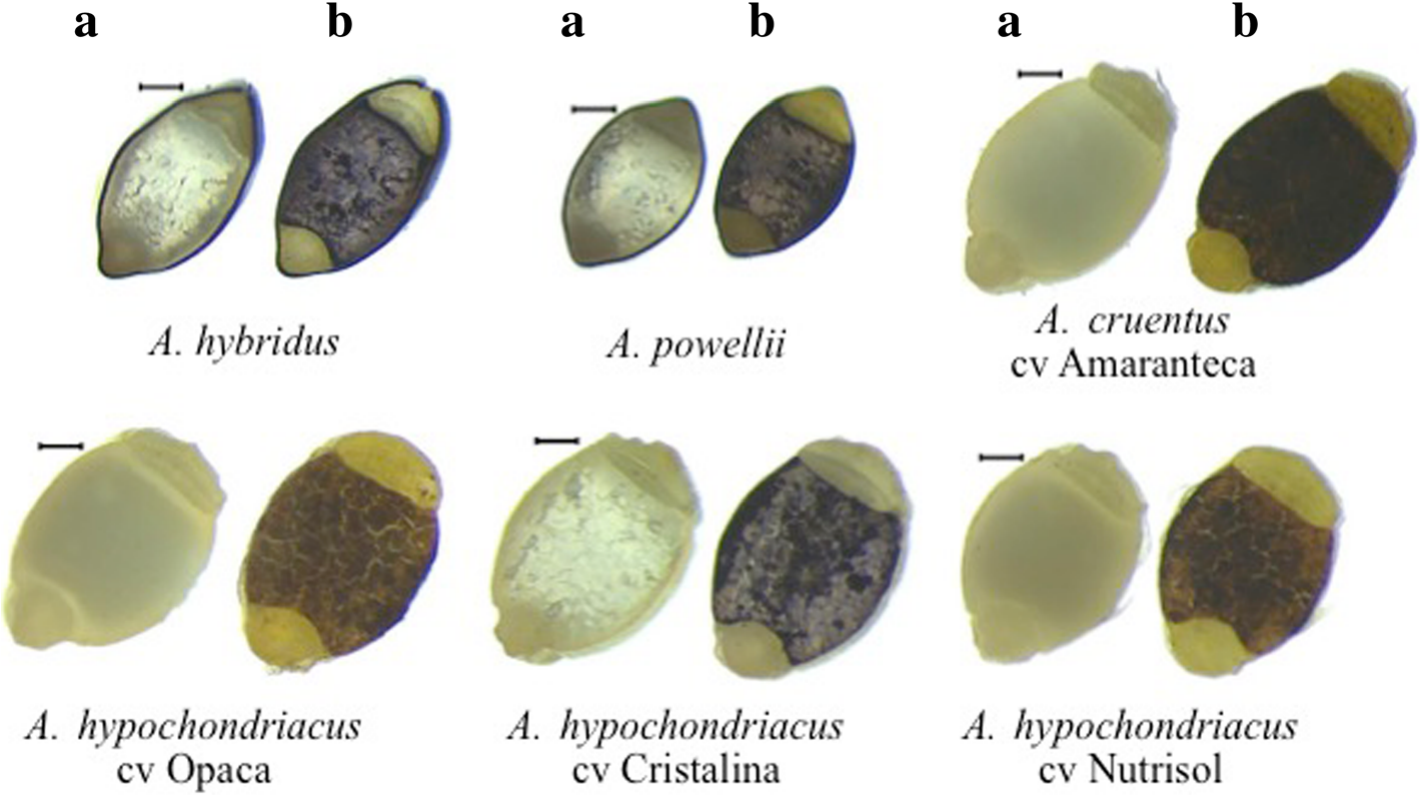
Transversal cuts of seeds from wild and cultivated amaranth species before (**a**) and after (**b**) iodine staining. Bars 200 μm

**Fig. 3:**
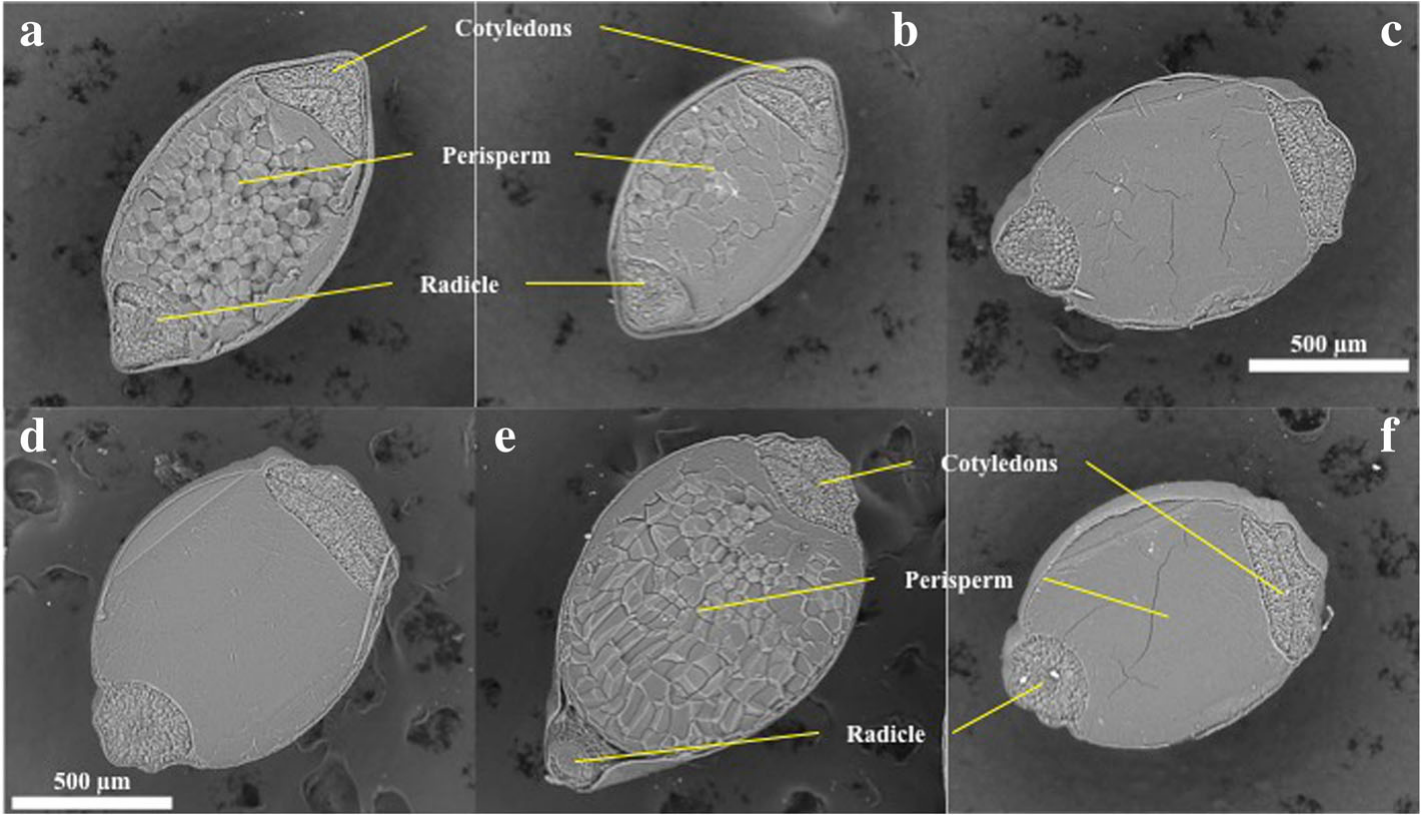
Scanning electron microscopy (SEM) images of transversal cuts of amaranth seeds. **a**, * A. hybridus*, **b**, * A. powellii*, **c**, * A. cruentus* cv Amaranteca, **d**, * A. hypochondriacus* cv Opaca (waxy), **e**, * A. hypochondriacus* cv Cristalina (non-waxy) and **f**, * A. hypochondriacus* cv Nutrisol

Phenotypic differences in amaranth seeds, which are characteristic of each species, were observed. Wild species are bright black seeds, while seeds of cultivated species are cream (Fig. 1). *A. powellii* contains the smallest seeds while *A. hybridus* and *A. cruentus* are the largest ones. Seeds crosscuts showed that the wild species *A. hybridus* and *A. powellii* are translucent; the cultivated species *A. cruentus* has opaque seeds while *A. hypochondriacus* cultivars were distinguished due to their translucent and opaque characteristics (Fig. 2a). Seeds iodine staining highlighted the structures within the starch perisperm (Fig. 2b). Wild species and *A. hypochondriacus* cv Cristalina stained purple-blue corresponding to non-waxy lines with high amylose content, while the opaque species stained red-brown corresponding to waxy lines with low amylose content. Seeds cross-sections were observed by SEM microscopy (Fig. 3) showing that in fact, *A. hybridus*, *A. powellii*, and *A. hypochondriacus* cv Cristalina have polyhedral structures in the perisperm, whereas the perisperms of *A. cruentus* cv Amaranteca, *A. hypochondriacus* cv Nutrisol and *A. hypochondriacus* cv Opaca did not show the typical polyhedral structure of amaranth starch granules.

### Protein hydrophobic fraction impacts on protein content and electrophoretic profile

**Fig. 4:**
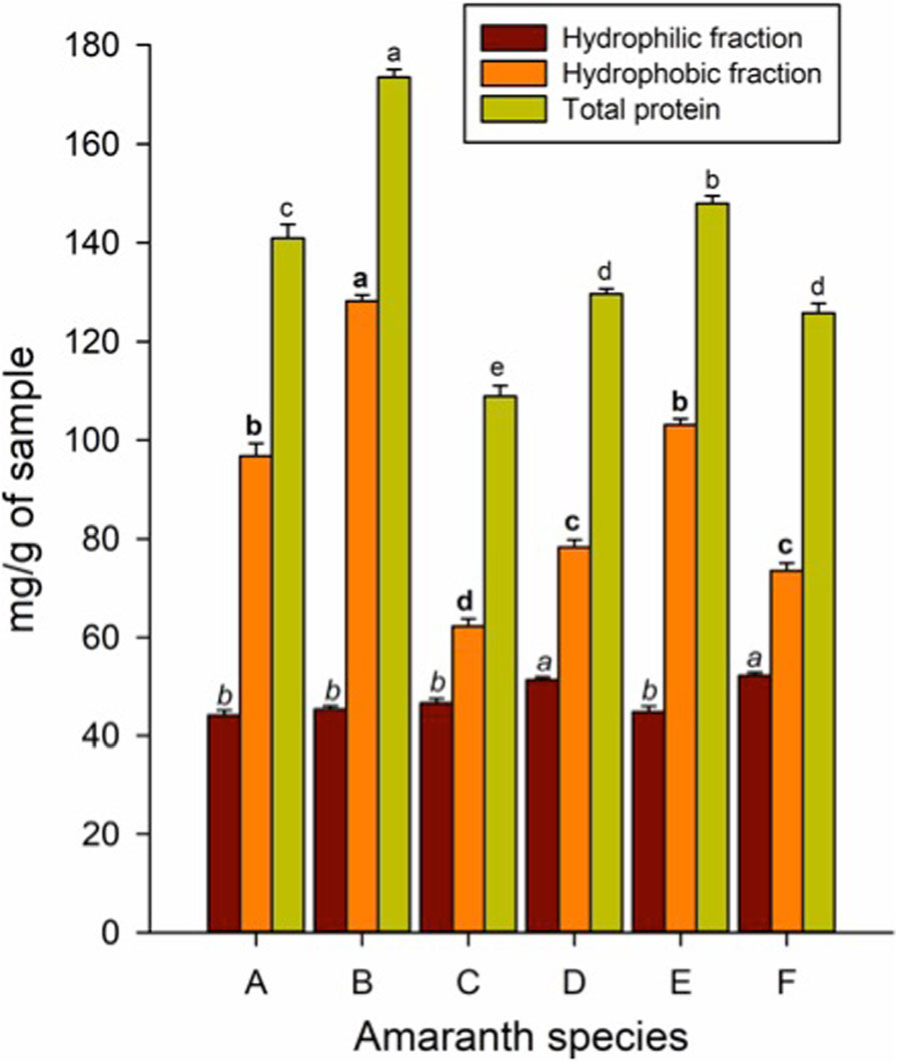
Bradford protein quantification of hydrophilic and hydrophobic proteins extracted from flour of wild and domesticated amaranth species. Protein quantification was carried out using the Bradford method. **a**, * A. hybridus*; **b**, * A. powellii*; **c**, * A. cruentus* cv Amaranteca; **d**, * A. hypochondriacus* cv Opaca (waxy); **e**, * A. hypochondriacus* cv Cristalina (non-waxy); **f**, * A. hypochondriacus* cv Nutrisol. Different letter above the bars indicates statistically differences at *p*< 0.05

In order to achieve greater coverage of seed proteins for analysis, extraction was carried out using a sequential approach based on protein polarity [27]. Results showed that *A. hypochondriacus* cvs Opaca and Nutrisol had more hydrophilic proteins (Fig. 4). However, differences in total protein content is reflected by the amount of hydrophobic protein fraction, hence that *A. powellii* has the highest protein content (173.5 mg/g), followed by *A. hypochondriacus* cv Cristalina and *A. hybridus* (147.9 and 140.8 mg/g, respectively). *A. cruentus* was the species with the lowest total protein content (108.8 mg/g).

**Fig. 5:**
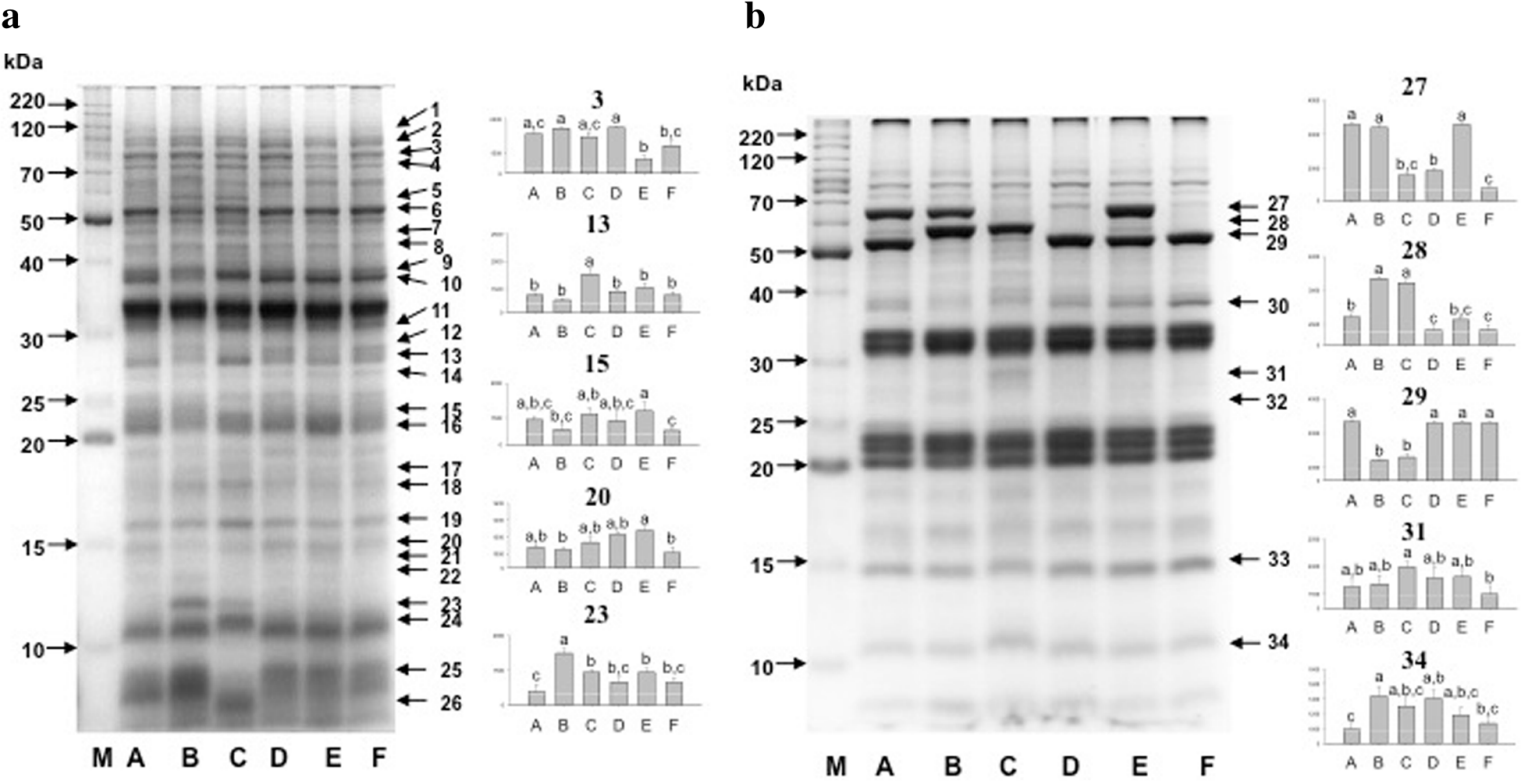
1D-SDS-PAGE profile of amaranth seed proteins. **a**, Hydrophilic proteins, **b**, Hydrophobic proteins. Lanes: M, molecular weight marker; A, *A. hybridus*; B, *A. powellii*; C, *A. cruentus* cv Amaranteca; D, *A. hypochondriacus* cv Opaca (waxy); E, *A. hypochondriacus* cv Cristalina (non-waxy); F, *A. hypochondriacus* cv Nutrisol. Arrows indicate the differentially accumulated protein bands selected for nLC-MS/MS identification. Densitometric analyses from selected bands are shown in graphics. Different letter in bands indicates statistically differences at *p* < 0.05

Electrophoretic profile of the hydrophilic fractions showed protein bands throughout all the separation range from below 10 kDa to above 220 kDa (Fig. 5a, Additional file 1: Figure S1). The most intense bands were observed at 33, 37, and 52 kDa. In contrast, the hydrophobic fraction showed lower number of bands, which were represented mainly by three groups, one between 20 to 24 kDa, the second from 32 to 35 kDa, and the last group, a highly variable region was formed with bands from 50 to 70 kDa (Fig. 5b, Additional file 1: Figure S2). In this fraction the presence or absence of bands (marked with a black arrow) amongst species was more evident than in the hydrophilic fraction. The histograms represent the differences in accumulation of some selected protein bands.

### Differentially accumulated proteins reflect the relationships amongst amaranth species

**Fig. 6:**
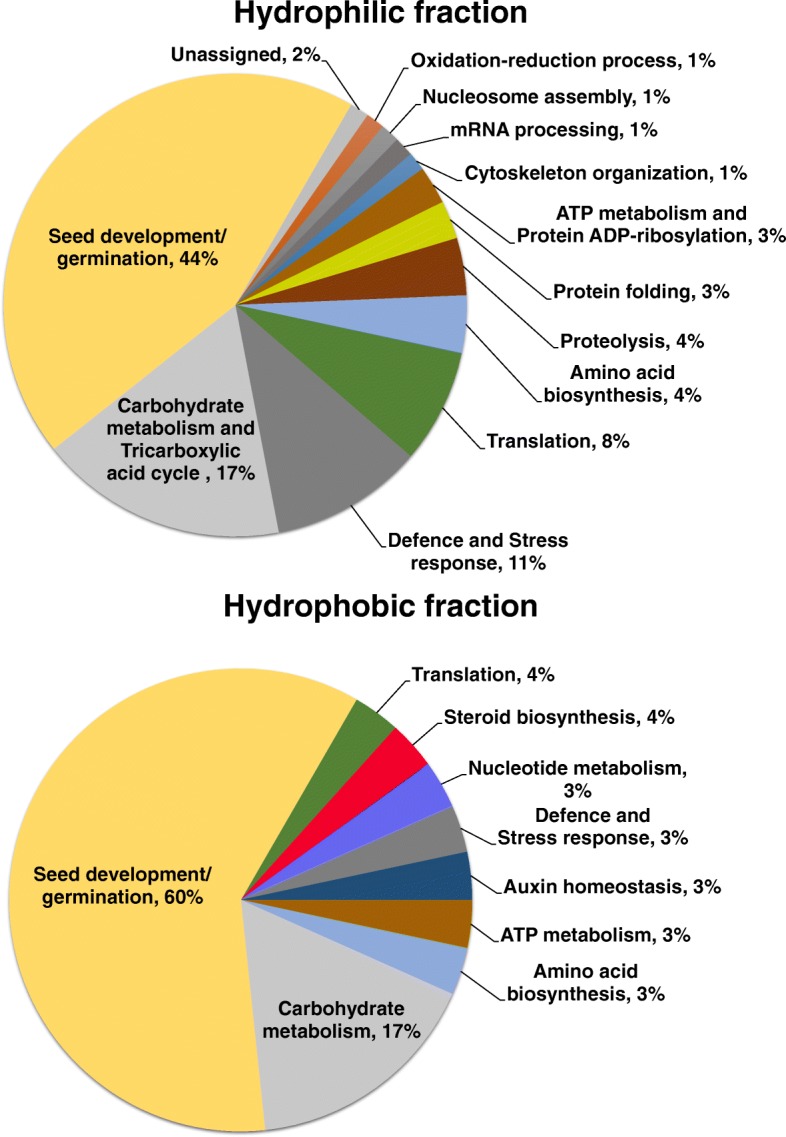
Classification of the proteins identified by nLC-MS/MS. The pie charts show the distribution into their biological process in percentage according to Gene Ontology Classification

Differentially accumulated protein bands were excised from gels (Fig. 5) and successfully identified by nLC-MS/MS (Table 1, Additional file 2: Table S1). In most of the cases more than one protein was identified in one band. The identified proteins were classified according to the Gene Ontology (GO) biological process annotation. In the hydrophilic fractions the differentially accumulated proteins were related with several functions being seed development and germination, carbohydrate metabolism, and response to stress and defence the most abundant (Fig. 6a). The differentially accumulated protein bands in the hydrophobic fraction were represented by proteins related with seed development and germination, carbohydrate metabolism, biosynthesis of amino acids, steroids, and auxin homeostasis (Fig. 6b).

**Table 1:** Amaranth proteins identified in differentially accumulated bands by nLC-MS/MS

Band^a^ No.	Protein	Acc. No.^b^	Mr^c^ Exp.	Mr^d^ Theor.	Mascot Score^e^	PM/SC^f^ (%)	emPAI^g^
1	Elongation factor 2	001926	97.28	94.87	116	8/11	0.30
	**Ribonuclease TUDOR 1**	**004841**		**109.14**	**58**	**18/20**	**0.38**
	Low-temperature-induced-like	018897		87.08	51	13/20	0.22
	Phosphoenolpyruvate carboxylase 3	004468		108.39	46	5/6	0.08
2	Alpha-xylosidase 1	020003	87.98	93.92	233	9/11	0.30
	Poly [ADP-ribose] polymerase 3	003773		82.76	166	12/23	0.29
	**Starch branching enzyme I**	**000673**		**100.56**	**160**	**16/20**	**0.41**
	Elongation factor 2	001926		94.87	132	10/17	0.34
	Chaperone 1	008070		100.77	81	9/12	0.19
	Aminopeptidase M1	006828		95.06	58	5/7	0.12
3	Methionine synthase	017360	78.85	78.493	529	18/30	0.39
	**Late embryogenesis abundant protein**	**013747**		**72.37**	**304**	**13/23**	**0.47**
	Methionine synthase	022179		89.82	219	9/16	0.17
	Sucrose synthase	021141		69.92	124	8/17	0.28
	Alpha-xylosidase 1	010666		100.26	111	7/9	0.19
	Disulfide isomerase	015532		56.01	51	5/14	0.13
4	**Vicilin-like**	**018839**	72.74	**61.21**	**191**	**8/18**	**0.25**
	**Embryonic protein DC-8-like**	**000638**		**65.20**	**61**	**6/10**	**0.23**
5	11S globulin	021282	57.85	78.10	186	16/32	0.31
	**Vicilin-like**	**018839**		**61.21**	**163**	**8/18**	**0.50**
	Catalase	007232		52.51	112	6/12	0.31
	Glucose-6-phosphate isomerase	013135		57.20	80	5/12	0.28
6	**Vicilin-like**	**018839**	52.81	**61.21**	**409**	**14/31**	**1.07**
	11S globulin	021282		78.10	356	18/34	0.69
	Late embryogenesis abundant protein	001171		45.86	265	8/24	0.95
	UTP-glucose-1-phosphate uridylyltransferase	008585		48.70	176	9/25	0.63
	**Enolase 1**	**001183**		**45.11**	**156**	**9/29**	**1.13**
	ATP synthase subunit mitochondrial-like	001716		59.31	145	7/15	0.50
	Adenosylhomocysteinase 1	009349		53.89	119	8/16	0.56
	Leucine aminopeptidase 1-like	014952		63.04	43	5/10	0.18
7	**Vicilin-like**	**018839**	**47.21**	**61.21**	**96**	**3/5**	**0.37**
	Eukaryotic initiation factor 4A-9	003448		47.07	45	3/9	0.15
8	Phosphoglycerate kinase	006883	43.28	87.94	573	19/27	0.51
	**Phosphoglycerate kinase**	**019107**		**42.55**	**379**	**14/38**	**1.13**
	11S globulin	021282		78.10	140	14/25	0.15
	Vicilin-like	018839		61.21	133	6/13	0.19
	Actin-7	019031		41.93	104	7/19	0.40
9	Glyceraldehyde-3-phosphate dehydrogenase	011043	38.41	31.58	410	9/37	0.44
	**Vicilin-like**	**018839**		**61.21**	**403**	**10/22**	**0.46**
	Aldose 1-epimerase-like	015176		32.27	132	6/24	0.27
	Dehydrin Rab18-like	003168		26.47	58	5/27	0.33
	11S globulin	021282		78.10	37	5/10	0.10
10	**Vicilin-like**	**018839**	37.42	**61.21**	**437**	**13/25**	**0.37**
	Lactoylglutathione lyase	011906		30.92	143	4/14	0.28
	Malate dehydrogenase	021284		36.13	124	6/29	0.19
11	Vicilin-like	006304	31.21	62.07	534	15/34	0.47
	Late embryogenesis abundant protein (SMP)	006906		28.70	190	6/33	0.60
	**Late embryogenesis abundant protein (SMP)**	**016810**		**22.64**	**167**	**5/34**	**1.10**
	11S globulin	001411		55.75	130	4/10	0.13
	11S globulin	021282		78.10	108	7/16	0.09
	60S ribosomal protein L6–3	005418		25.60	94	5/29	0.70
	Agglutinin	007409		30.40	80	4/18	0.56
12	**Oil body-associated protein 1A**	**009953**	29.19	**26.80**	**255**	**11/35**	**1.22**
	Vicilin-like	006304		62.07	186	3/9	0.12
	60S ribosomal protein L7–4	008528		28.35	180	5/18	0.65
	Elongation factor 1-beta 1	002577		24.87	170	3/20	0.53
	**Oil body-associated protein 2A**	**004342**		**25.76**	**98**	**7/29**	**0.99**
	Protein synthesis inhibitor PD-S2-like	011528		30.52	94	4/19	0.42
13	**Protein synthesis inhibitor PD-S2-like**	**011528**	27.81	**30.52**	**362**	**13/42**	**1.39**
	Oil body-associated protein 2A	004342		25.76	193	6/25	0.80
14	**Cysteine proteinase inhibitor 6**	**021786**	26.87	**27.78**	**270**	**11/52**	**1.44**
	Vicilin-like	018839		61.21	76	5/11	0.26
	11S globulin	021282		78.10	46	5/10	0.15
**15**	**11S globulin**	**001411**	**22.98**	**55.75**	**377**	**8/19**	**0.47**
16	**11S globulin**	**021282**	21.73	**78.10**	**290**	**10/16**	**0.51**
	11S globulin	001411		55.75	242	5/12	0.38
17	**17.6 kDa class I heat shock protein 3**	**013876**	17.93	**17.92**	**182**	**7/46**	**1.18**
	**Oleosin 5**	**013707**		**20.73**	**72**	**5/28**	**0.96**
	Oleosin 5	015343		20.46	69	4/21	0.67
18	Cyclophilin	002428	17.43	17.21	85	2/13	0.26
	17.4 kDa class I heat shock protein 3	012223		17.36	34	6/33	0.25
19	**Late embryogenesis abundant protein (LEA_5)**	**008005**	**15.82**	**9.66**	**152**	**5/59**	**2.50**
20	Vicilin-like	018839	14.82	61.21	84	4/8	0.30
23	Histone H4	005348	11.74	11.40	62	5/41	1.51
**24**	**Late embryogenesis abundant protein (LEA_5)**	**019862**	**10.73**	**8.53**	**49**	**3/49**	**0.47**
27	**Granule-bound starch synthase chloroplastic amyloplastic**	**011500**	63.25	**63.00**	**1112**	**27/58**	**3.08**
	11S globulin	021282		78.10	287	14/26	0.42
	Vicilin-like	018839		61.21	231	10/23	0.48
	Indole-3-aceticacid-amido synthetase	011444		70.17	65	4/6	0.21
28	**Granule-bound starch synthase chloroplastic amyloplastic**	**011500**	56.53	**63.00**	**837**	**19/43**	**2.36**
	11S globulin	021282		78.10	475	17/38	1.04
	Vicilin-like	018839		61.21	38	3/6	0.12
29	**11S globulin**	**021282**	52.41	**78.10**	**412**	**20/43**	**1.22**
	Granule-bound starch synthase chloroplastic amyloplastic	011500		63.00	122	8/13	0.47
	Vicilin-like	018839		61.21	119	10/23	0.66
	ATP synthase subunit mitochondrial-like	001716		59.31	116	9/21	0.59
	Late embryogenesis abundant protein	001171		45.86	114	5/13	0.35
	Elongation factor 1-alpha 1	001308		50.96	66	7/17	0.50
	Serine hydroxymethyltransferase 4	009350		59.74	66	4/12	0.12
30	Vicilin-like	018839	37.95	61.21	333	9/23	0.50
	11S globulin	021282		78.10	210	13/23	0.50
	Aldose 1-epimerase-like	015176		32.27	140	5/21	0.38
	11-beta-hydroxysteroid dehydrogenase 1B	004692		74.56	109	9/18	0.21
	**Glyceraldehyde-3-phosphate dehydrogenase**	**013553**		**31.71**	**83**	**6/28**	**0.73**
31	Vicilin-like	006304	19.47	62.10	90	7/17	0.20
	**Oil body-associated protein 1A**	**009953**		**26.79**	**89**	**7/35**	**1.60**
32	Vicilin-like	006304	27.11	62.10	90	3/5	0.06
	**Protein synthesis inhibitor PD-S2-like**	**011528**		**30.52**	**82**	**8/30**	**0.81**
	Oil body-associated protein 2A	004342		25.76	45	4/20	0.32
33	Vicilin-like	018839	14.75	61.21	137	4/9	0.27
	Vicilin-like	006202		59.16	109	5/7	0.28
	Nucleoside diphosphate-kinase1	014404		16.19	45	3/17	0.22
	**11S globulin**	**001411**		**55.75**	**42**	**3/6**	**0.55**
34	**11S globulin**	**001411**	10.72	**55.75**	**109**	**6/15**	**0.22**
	**Vicilin-like**	**006202**		**59.16**	**54**	**5/7**	**0.28**

^a^Band numbers according Fig. 5. ^b^Accession number according to the database reported by Clouse et al. [9]. ^c^Experimental molecular weight (kDa). ^d^Theoretical molecular weight (kDa). ^e^MASCOT Score, individual ion scores > 33 are statistically significant (*P* < 0.01), only identifications with peptide matches above identity threshold when FDR ≤5% were considered true. ^f^Peptides Matched/Sequence Coverage. ^g^Exponentially Modified Protein Abundance Index. Protein names in bold letters are discussed in the text

**Fig. 7:**
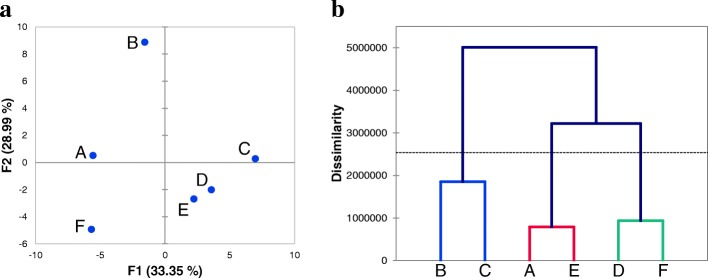
Principal Components Analysis (PCA) and Agglomerative Hiererchical Clusterin (AHC). **a**, Principal component score plot for the data set. The first two components account for 62.34% of the total variation. Each axis is labelled with the percent of total variance and the absolute eigenvalue. **b**, AHC dendogram grouped amaranth species according to their similarity on protein profiles. Letters correspond to amaranth species: A, *A. hybridus*; B, *A. powellii*; C, *A. cruentus* cv Amaranteca; D, *A. hypochondriacus* cv Opaca (waxy); E, *A. hypochondriacus* cv Cristalina (non-waxy); F, *A. hypochondriacus* cv Nutrisol

With the information of protein content in seeds and the differentially accumulated bands intensity, PCA (Principal component analysis) and AHC (Agglomerative hierarchical clustering) analyses were carried out. PCA maps showed that two principal components accounted for 63.34% of variation (Fig. 7a). These two main components grouped the wild species in the same quadrant, *A. cruentus* was located alone in one quadrant near to *A. hypochondriacus* (Opaca and Cristalina) and the most cultivated species *A. hypochondriacus* cv Nutrisol was the most distant from the rest of the species. The AHC dendrogram clearly indicates that *A. powellii* and *A. cruentus* have a close relationship as well as *A. hybridus* and *A. hypochondriacus* cv Cristalina (Fig. 7b).

### LEA proteins are species-specific

Different paralogs of late embryogenesis abundant proteins (LEAs) were identified (Table 1, Additional file 1: Table S2). In band 3, which was down accumulated in *A. hypochondriacus* cv Cristalina, was detected one LEA (AHYPO_013747); in band 4 (up accumulated in *A. powellii and A. cruentus*) was detected the Embryonic DC-8 like (AHYPO_000638), and in band 6, which was observed accumulated in *A. hybridus* and diminished in *A. powellii*, the LEA (AHYPO_001171) was detected. Two LEA proteins (AHYPO_006906 and AHYPO_016810) containing the Seed Maturation Protein (SMP) motif were identified in band 14, whose accumulation decreased in wild species. In bands 19 and 24, from *A. cruentus* and *A. powellii*, was identified only one protein corresponding to LEA AHYPO_008005 and AHYPO_019862, respectively. These two proteins showed the LEA_5 domain, which is one of the most hydrophilic LEAs [28]. Interestingly the previously characterized AcLEA protein (AHYPO_005092), was not detected in any differentially accumulated protein band, which is in agreement with the observation that this LEA is very conserved among wild and cultivated amaranth species [29].

### Differential accumulation of GBBSI and oil bodies related proteins amongst species

The most striking differences in protein profiles among amaranth species were detected in the hydrophobic fraction, especially in bands 27, 28, and 29 (Fig. 5b, Table 1). In those bands, different proteoforms of the granule-bound starch synthase I (GBSSI, AHYPO_011500) were identified. The accumulation of band 27 only in wild species (*A. hybridus* and *A. powellii*) as well as in *A. hypochondriacus* cv Cristalina, correlates with the observation that these species are classified as non-waxy type (Fig. 2). However band 28 is representative of *A. powellii* and *A. cruentus* cv Amaranteca, which are non-waxy and waxy phenotypes, respectively. By contrary band 29 was detected in *A. hybridus* as well as in all *A. hypochondriacus* cultivars. As observed, only the GBSSI of higher molecular weight (band 27) correlates with the non-waxy phenotype (Figs. 2 and 3), thus this protein could be the functional waxy enzyme.

In band 17, up accumulated in *A. cruentus*, were identified two paralogs of oleosin 5 (AHYPO_013707 and AHYPO_015343). Accumulation of band 12 was observed in *A. hybridus* and *A. powellii*, in this band was identified two paralogs of oil body associated proteins (OBAPs). OBAP1 (AHYPO_009953) and OBAP2 (AHYPO_004342); while in protein band 13 more accumulated in *A. cruentus* was detected another OBAP2. A vicillin isoform was also identified in band 12, which is in agreement with Zhao et al. [30], who reported that during oil body extraction in soybean, glycinin and β-conglycinin are co-purified.

### Identification of new paralogs of amaranth globulins

Different paralogs of 7S and 11S globulins were detected in different protein bands (Table 1**)**. The canonical 7SB (AHYPO_006304) containing the β-barrel or cupin structural domain, which function as nutrient reservoir, was detected down-accumulated in wild species (band 11) as well as in *A. hypochondriacus* cv. Nutrisol (band 31). The vicilin, containing antimicrobial peptide domain (AHYPO_006202), was accumulated in *A. hybridus* (band 33) and *A. powellii* and *A. cruentus* (band 34). The 7SD globulin (AHYPO_18839) containing both cupin and vicilin domains, was identified preferentially accumulated in *A. powellii* and *A. cruentus* (bands 4 to 12, and 14) as well as in *A. hypochondriacus* cv Cristalina and cv Nutrisol (bands 20, 30, and 33). The presence of this protein in different molecular weights could be explained by posttranslational proteolytic processing during the deposition and storage process [31].

The 11S globulin Ah11SB (AHYPO_001411) accumulated less in *A. hybridus* than in *A. powellii* (band 34) but more in *A. hypochondriacus* cv Cristalina (band 15). The legumin (AHYPO_021282), named as Ah11SHMW due to its unusual high molecular weight, was found more accumulated in *A. hybridus* and *A. hypochondriacus* (band 29)*.* A fourth 11S globulin, named Ah11SPheRich (AHYPO_006768), was found by searching in the proteome database, but it was not differentially accumulated amongst amaranth species.

**Fig. 8:**
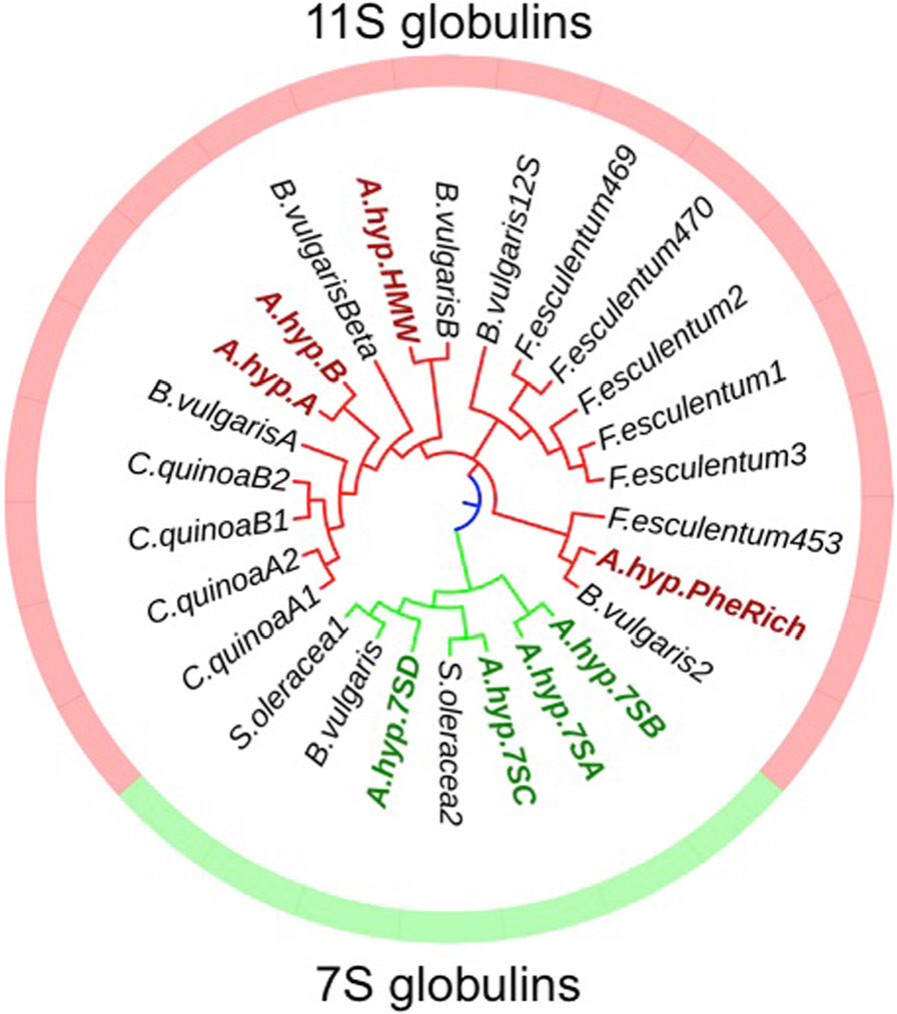
Phylogenetic relationships of seed storage proteins belonging to the cupin superfamily of the order Caryophyllales. Phylogenetic tree was constructed with the neighbour-joining method and a bootstrap test for 1000 replicates. Red arrows indicate 7S and 11S amaranth globulins. Sequences names and NCBI or Phytozome identification numbers: *B. vulgaris* (XP_010679084.1); *S. oleracea* 1 (XP_021843200.1); *S. oleracea* 2 (XP_021861035.1); *A. hyp* A (3QAC_A); *A. hyp* B (AHYPO_001411-RA); *A. hyp* PheRich (AHYPO_006768-RA); *A. hyp* HMW (AHYPO_021282-RA); *C. quinoa* A1 (AAS67036.1); *C. quinoa* A2 (ABI94735.1); *C. quinoa* B1 (AAS67037.1); *C. quinoa* B2 (XP_021770181.1); *B. vulgaris* Beta (XP_021770181.1); *B. vulgaris* 2 (XP_010679299.1); *B. vulgaris* A (XP_010679302.1); *B. vulgaris* B (XP_010671027.1); *B. vulgaris* 12S (XP_010671026.1); *F. esculentum* 1 (O23878.1); *F. esculentum* 2 (O23880.1); *F. esculentum* 3 (Q9XFM4.1); *F. esculentum* 453 (AAP15457.1); *F. esculentum* 470 (BAO50869.1); *A. hyp* 7SA (AHYPO_010140-RA); *A. hyp* 7SB (AHYPO_006304-RA); *A. hypochondriacus* 7SC (AHYPO_007944-RA); *A. hypo* 7SD (AHYPO_018839-RA)

The phylogenetic tree constructed with 7S and 11S globulins from amaranth and members from other Caryophyllales belonging to the cupin superfamily, which is characterized by the presence of β-barrel structural domains [32], revealed that Ah11SA and Ah11SB are very close, however Ah11SHMW and AhPheRich are more similar to *Beta vulgaris* orthologs and it is very clear that 7S globulins formed another branch on the tree (Fig. 8).

### In silico molecular characterization of amaranth 11S globulins paralogs

**Fig. 9:**
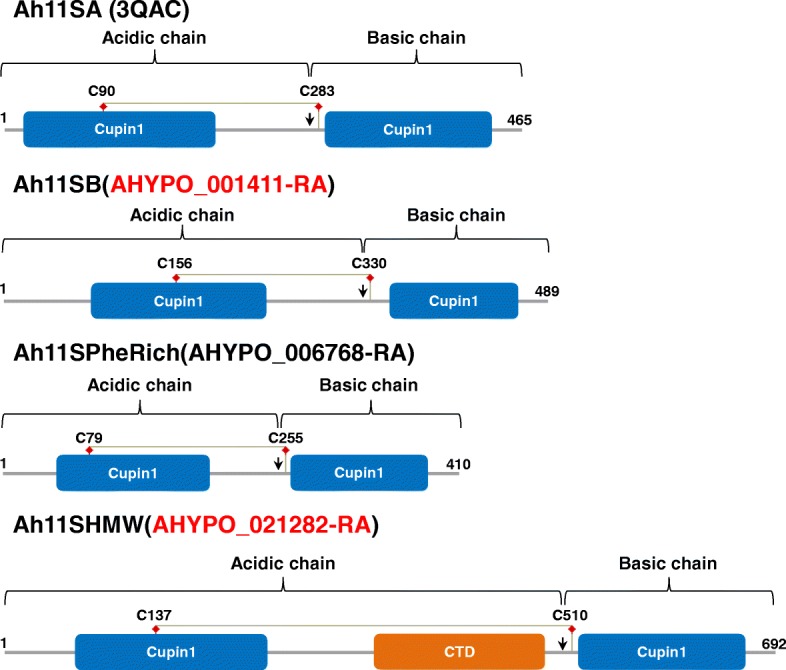
Conserved domains in amaranth 11S globulins. All monomers have two cupin domains. Cysteine residues involved in the formation of the disulfide bond between the acidic and the basic subunits are indicated. The arrow in each diagram indicates the proteolytic processing Asn-Gly site to which 11S globulins are subjected during its synthesis and deposition, giving rise to the subunits

Clustal analysis for amaranth 11S globulins compared against the canonical and well-known soybean 11S globulins was carried out (Additional file 1: Figure S3). All globulins present highly conserved structural features, as the proteolytic site Asn-Gly that is cleaved by a specific asparaginil endopeptidase generating the acidic and basic subunits linked by disulphide bonds, each one containing a cupin *b*-barrel domain (Additional file 1: Figure S4). However, some differences in structure were observed when compared with the canonical Ah11SA (Fig. 9). Ah11SB has a larger acidic chain and a short basic chain. Globulin denominated as Ah11SPheRich because at primary structure level shows high percentage of Phe (17.1%) in comparison with the other globulins (2.8 to 5.2%) (Additional file 1: Figure S5). The Ah11SHMW is a globulin paralog of high molecular weight showing the largest acidic chain (Fig. 9). The analysis of Ah11SHMW primary structure showed a segment of 18 amino acid residues: G-S-E(Q)-W(R)-D(E)-P-R(S)-Y-P-G-H-G(E)-S-Q(E)-R-P-A(G/T)-H that is repeated 9 times within the acidic subunit (Additional file 1: Figure S6). This segment was identified in SMART and Pfam servers as CTD domain, which is known to be involved in the regulation of transcript elongation process and mRNA processing, but until now, there are no reports about an 11S globulin containing this domain neither about its biological function.

**Fig. 10:**
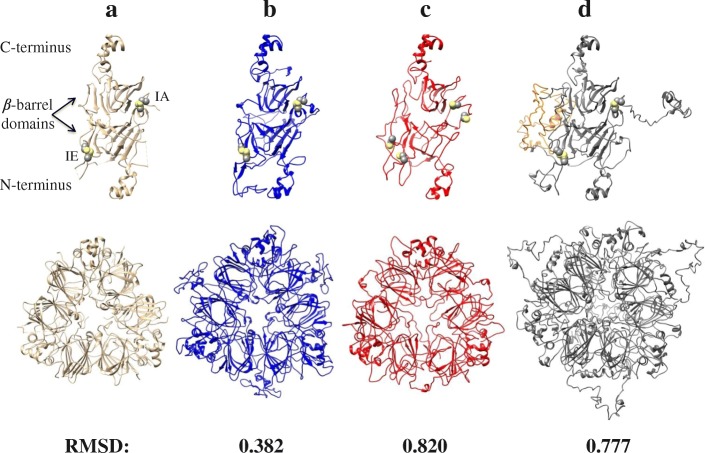
**a**, Experimental reported structure for the canonical 11S globulin monomer of *A. hypochondriacus* (Ah11SA, PDB 3QAC) and structural models generated from 11S globulin paralogs sequences. **b**, Ah11SB (001411); **c**, Ah11SPheRich (006768); **d**, Ah11SHMW (021282). The low RMSD values indicate that all globulins are structural homologues. All globulins present the two β-barrel domains characteristic of these proteins, the highly conserved cysteines are shown in yellow spheres, which are involved in the formation of intra- (IA) and inter-chain (IE) disulphide bonds. The orange region in the model of Ah11SHMW delimits the CTD-like domain exclusive of this paralog, which is not present in any other 11S globulin reported so far

**Fig. 11:**
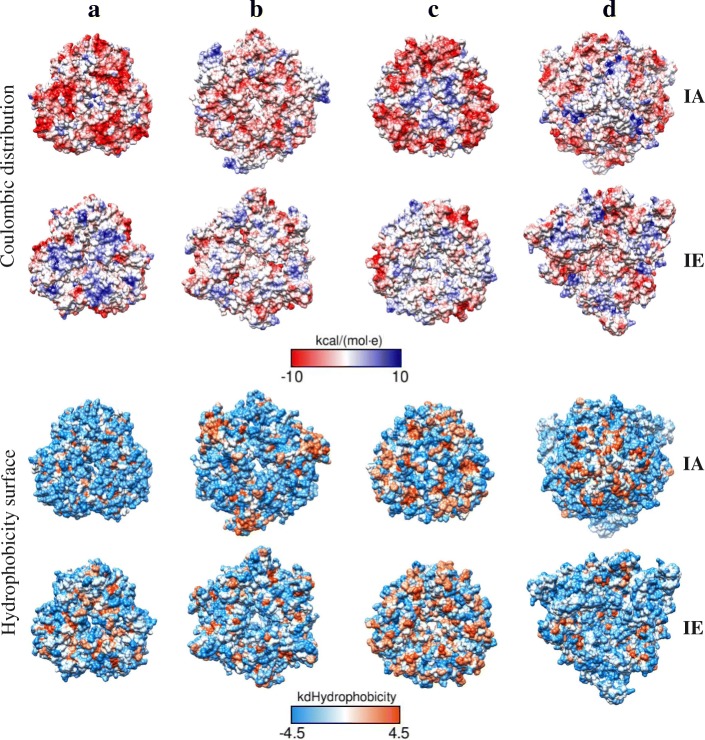
Coulombic distribution and hydrophobicity surface of IA and IE faces trimeric structures of 11S globulins paralogs from *A. hypochondriacus*. **a**, Ah11A, **b**, Ah11SB (001411), **c**, Ah11SPheRich (006768), **d**, Ah11SHMW (021282)

In amaranth only the canonical 11S globulin, one of the most abundant proteins in the hydrophobic fraction, has been characterized at structural level by X-ray crystallography and named Ah11SA with PDB identifier 3QAC [33] (Fig. 10a). Three-dimensional structures of all amaranth 11S globulin paralogs were generated by homology modelling and compared with Ah11SA. The models presented the β-barrel and α-helices distinctive domains of legumin monomers (Fig. 10b, c and d). When compared with Ah11SA, the RMSD values for Ah11SB, Ah11SHMW and Ah11SPheRich were of 0.382, 0.777, and 0.820, respectively, indicating that these proteins are structural homologs. Yellow circles in models represent the intra- (IA) and inter- (IE) chain disulphide bonds. The orange non-structured region in Ah11SHMW represented the highly exposed CTD-like domain. The hydrophobicity and coulombic surfaces of both faces (IA and IE) of amaranth globulins structures are shown in Fig. 11. 11S globulins hydrophobic residues are located mainly on the central part of the IA face (orange region), but the hydrophobicity surface changes among the distinct paralogs being the Ah11SPheRich the more hydrophobic which correlates with its high Phe content.

## Discussion

Amaranth has greatly gained attention due to its agronomical and nutraceutical characteristics. However, only a few species, from various available, are cultivated for seeds production. Amaranth wild relatives have survived for thousand years growing under different environments such as very saline soils, high temperatures, UV radiation, and water deficit [26]. Accordingly, they are considered important reservoirs of useful genes/proteins involved in plant resistance [24, 25]. However, information about morphological and molecular characteristics of wild amaranth species have not been reported.

Although *A. powellii* produces the smallest seed, this is the species with the highest protein content, while *A. cruentus*, one of the cultivated species, is the one with the lowest values. Thus, *A. powellii* represents an interesting option as a source of information that could be used to increase protein content in cultivated ones. Similar results have been reported for rice species (*Oryza* spp.) indicating that wild species contained higher protein amounts than the domesticated species, differences that were attributed to the glutelins fraction [34]. It is also known that glutelins in amaranth are an important seed storage protein fraction accounting for 23 to 42% of the total seed protein, depending on the extraction conditions [35]. The group of bands between 50 and 70 kDa has previously been detected as differentially accumulated in varieties of cultivated amaranths. Consequently, this protein fraction was suggested as a tool for identification of amaranth accessions [36, 37].

In orthodox seeds, LEA proteins have been associated with desiccation tolerance and maintenance in a quiescent state. LEAs are classified on the basis of amino acid sequence and conserved motifs into five to nine sub-classes [38]. A good correlation between the abundance of certain LEAs and seed longevity has been reported [5, 39]. By searching in amaranth database, 39 LEA protein sequences with particular motifs were identified (Additional file 1: Table S2), but only some of them were found differentially accumulated amongst species. The Embryonic DC-8 and LEA_5 group were detected preferentially accumulated in *A. powellii* and *A. cruentus*. DC-8 protein has been detected during embryogenesis and in cell walls of endosperm tissues, however its function is still unclear [40, 41]. LEA_5 and SMPs are proteins related with water stress tolerance [42], SMP was less accumulated in wild species (*A. powelli* and *A. hybridus*) but preferentially accumulated in cultivated species, this is interesting since *A. cruentus* contain both LEA_5 and SMP proteins and is one species that can grow under severe water deficit [25, 26].

OBAPs (bands 13, 31, 32) as well as two paralogs of olesins (band 17) were more abundant in *A. cruentus*. It has been shown that OBAPs are involved in oil bodies biogenesis, stability, trafficking, and mobilization [43]. Oleosins act as natural emulsifiers and protect plant lipid reserves against oxidation and hydrolysis until seed germination and seedling establishment [44]. The putative role of some oleosins is related to controlling lipid body size and maintenance of its integrity [45]. It has been reported that an *A. thaliana* mutant deficient in OBAP1 shows changes in fatty acid composition, reduction of germination rate, and seed triacylglycerols content [46]. Therefore, the differential accumulation of OBAP1 and OBAP2 could be related with the quantity and quality fat composition among amaranth species. These observations correlated with the relative abundance of fatty acids and hydrocarbons, such as squalene, reported for wild and cultivated amaranth species [47].

GBSSI, also known as waxy protein, is a glucosyltransferase and the only enzyme responsible for elongation of amylose polymers in nutrient storage tissues [48]. Park et al. [48], analysed the *Waxy* locus in amaranth showing that a nonsense mutation in the coding region at exon 6 in *A. cruentus* and exon 10 in *A. hypochondriacus* prematurely ends translation and causes complete loss of gene function, leading to a waxy phenotype. Then the GBSSI identified in bands 28 and 29 could correspond to the non-functional truncated enzyme. Ahuja et al. [49] reported that during wheat development, GBSSI considerably affects starch accumulation and glucan chain length distribution. It is known that high amylose contents could contribute to resistant starch (RS) through the formation of inclusion complexes with lipids [50]. Zhou et al. [51] have proposed a mechanism in which the deficiency in sucrose synthase III (SSIIIa) and the presence of GBSSI could be the responsible for RS accumulation.

SSPs are accumulated during seed development to serve as source of amino acids during germination and early seedling growth and represent the main source of protein for food and feed consumption. Globulins are the most abundant SSPs in dicotyledoneus plants and are classified in two groups based on their sedimentation coefficients in 7S or vicilins and 11S or legumins [31]. The *A. hypochondriacus* database contains 13 different 7S globulin protein sequences, with members belonging to the three different types, which are classified on the basis of their structural domains (Additional file 1: Table S3). Only three of them were differentially accumulated amongst amaranth species. The 7S containing the antimicrobial domain was representative in wild species as well as in *A. cruentus*, on the other hand the canonical cupin-type was more representative in *A. hypochondriacus* species.

11S globulins or legumins are the more widely distributed SSPs in nature and are encoded by multigenic families. The soybean 11S globulin or glycinin, is composed by five different monomers, each encoded by a different gene [52]. In amaranth only the canonical 11S globulin has been reported and characterized [53]. Here we have detected two more paralogs differentially accumulated among wild and cultivated amaranths (Table 1, Additional file 2: Tables S1). The CTD-like domain, identified by database searching in Ah11SHMWglobulin, has some special features: all of those repeats have conserved Ser and Tyr that could be involved in signalling process by phosphorylation; His and Arg, positively charged amino acids that affect de solubility and assembly of a protein depending of pH variations; and two Pro, amino acid known as secondary structure breaker. It is possible that this domain suffers some posttranslational modifications and has some biological activity in seeds, but further work should be done in this direction.

Recently the importance of SSPs has increased due to the presence of different paralogs and the fact that some of them do not only are nutrient reservoirs, but are also involved in other functions during seed development or germination [18]. A novel function for 11S globulins as auxin transporters have been reported, in which during the germination process, the change in pH induces the hexamer dissociation and its release, suggesting globulins as novel players in hormone homeostasis [54]. New roles of SSPs have been reported as buffer proteins against oxidative stress that might imply an important role in seed longevity [7, 16, 17].

The surface properties of a protein, mainly hydrophobicity and charge distribution are very important since they dictate the physicochemical functionality of the molecule [55]. Three-dimensional structure models of amaranth legumins showed similar features to the canonical 11S globulins, but they show some particular characteristics, variation in the superficial charged and hydrophobic residues distribution for example, which can confer differentiated functional properties to each legumin, like solubility or the ability to form interactions with other molecules. These physicochemical variations between amaranth 11S globulins paralogs are of relevance for two topics, first the application of the proteins as additives for the stabilization of food systems, and second, the implications in biological processes like seed development and germination.

## Conclusion

This is the first report of molecular characterization of wild amaranth species in comparison with cultivated ones. Seed electrophoretic patterns have been very powerful tool in detecting differential accumulation of several proteins amongst wild and cultivated species. It is interesting to highlight that protein accumulation profile indicates that *A. powellii* is more closely related to *A. cruentus*. LEAs could be potential targets for seed resistance and defence traits. OBAPs and oleosins could be target to increase squalene content in seeds. Overall our results suggest that there are many new types of globulins paralogs and precursors in wild species, thus, wild amaranth species are very important genetic resources for improving the nutritional quality of amaranth seeds. New paralogs of 11S globulins were detected and structurally characterized in silico. Further work is needed to understand the biological functions of the newly identified globulins in amaranth seeds.

## Materials and methods

### Plant materials

Four amaranth samples, two black-seeded wild species: *A. hybridus* and *A. powellii*, and two cream-seeded cultivated species, *A. cruentus* cv Amaranteca and three *A. hypochondriacus* cultivars: Cristalina, Opaca, and Nutrisol were used for analysis. Samples were kindly provided by the National Institute for Forest, Agricultural and Livestock Research (INIFAP, Mexico).

### Morphological and structural analysis of seeds

Images of whole seeds and cross-sections were obtained with the SteREO Discovery V8 (Carl Zeiss, Oberkoche, GE). Scanning electron microscopy images of amaranth seeds were captured with an ESEM model Quanta 200 (FEI, Hillsboro, OR, USA) from the National Laboratory of Nanosciences and Nanotechnology Research-IPICYT. Cross-sections were stained with an iodine solution (2% KI (*w*/*v*), 1% I_2_ (w/v) for 30 s, washed with distilled water for 1 min and observed at the stereoscope.

### Extraction of total protein from seed

Protein extraction was carried out according to Saucedo et al. [29]. Seeds were frozen in liquid nitrogen and ground in mortar and pestle. Flours were defatted with hexane in a 1:10 (w/v) ratio. The flour:hexane mixture was homogenized using vortex at maximum speed for 15 min at 4 °C, then centrifuged at 15,000×g for 30 min at 4 °C in a Beckman Avanti J-26S XPI centrifuge (Beckman, California, USA). The supernatant was discarded and the precipitate air-dried. Proteins of polar nature were extracted from the defatted flour using 0.1 M 2-amino-2-(hydroxyl-methyl)propane-1,3-diol, pH 8.5 containing 10% (*v*/v) glycerol and 2 mM PMFS (Sigma-Aldrich, St. Louis, MO, USA) at 1:20 (w/v) ratio. Mixture was agitated by vortex for 15 min at 4 °C and centrifuged at 17,000×g for 30 min at 4 °C. For extraction of hydrophobic proteins (including non-polar, membrane, and cell wall proteins), the residue resulting from the hydrophilic fraction was resuspended in a solution of 7 M urea, 2 M thiourea, 2% (w/v) CHAPS, 2% (v/v) Triton X-100, mixed and centrifuged as mentioned above. Protein concentration was determined using the Protein Assay reagent (Bio-Rad, Hercules, CA, USA), and bovine serum albumin as standard. All extractions and measurements were carried out in triplicates. Protein extracts of three independent biological replicates were applied to 1D-SDS-PAGE as described below.

### Electrophoretic profile of amaranth proteins

Protein extracts of hydrophilic and hydrophobic protein fractions were analysed by 1D-SDS-PAGE in discontinuous Tris-glycine gels using 4 and 13.5% of acrylamide final concentration for the stacking and resolving gels, respectively. Protein extracts (50 μg) from each sample were loaded and separated in a SE 600 Ruby chamber (GE Healthcare, Little Chalfont, Buckinghamshire, UK) at 10 mA/gel for 1 h followed by 25 mA/gel for 4 h. After electrophoresis, gels were stained with a 0.05% Coomassie Brilliant Blue R-250 (USB Corporation, Cleveland, OH, USA) in 40% methanolic solution containing 10% acetic acid and distained with the same solution without the dye. Gels were digitalized in a Gel Doc XR+ Imaging System apparatus (Bio Rad) and densitometry analysis was performed with Quantity One software v4.5 (Bio Rad).

### Statistical analysis

Densitometric data was submitted to an analysis of variance (ANOVA) with Holm-Sidak test using the Sigma Plot software v12.3 (Systat Software, Inc., San Jose, CA, USA), considering *p* < 0.05 for statistically significant differences. Bands with statistically different intensities for at least one species were selected for mass spectrometry analysis. Principal Component Analysis (PCA) and Agglomerative Hierarchical Clustering (AHC) were done using XLSTAT software (Addinsoft, Paris, France).

### In-gel digestion and mass spectrometry analysis

Differentially accumulated protein bands were excised from the 1D-SDS-PAGE, distained, reduced and alkylated as described by Huerta-Ocampo et al. [25]. Protein digestion was carried out overnight at 37 °C with sequencing-grade trypsin (Promega, Madison, WI, U.S.A.). Nanoscale LC separation of tryptic peptides was performed with a nanoACQUITY UPLC System (Waters, Milford, MA, USA) equipped with a Symmetry C18 precolumn (5 μm, 20 mm × 180 μm, Waters) and a BEH130 C18 (1.7 μm, 100 mm × 100 μm, Waters) analytical column. The lock mass compound, [Glu1]-Fibrinopeptide B (Sigma-Aldrich), was delivered by the auxiliary pump of the nanoACQUITY UPLC System at 200 nL/min at a concentration of 100 fmol/mL to the reference sprayer of the Nano-Lock-Spray source of the mass spectrometer. Mass spectrometric analysis (LC-MS/MS) was carried out in a SYNAPT-HDMS Q-TOF (Waters). The spectrometer was operated in V-mode, and analyses were performed in positive mode ESI. The TOF analyzer was externally calibrated with [Glu1]-Fibrinopeptide B from m/z 50 to 2422. The data were lock-mass corrected post-acquisition using the doubly protonated monoisotopic ion of [Glu1]-Fibrinopeptide B. The reference sprayer was sampled every 30s. The RF applied to the quadrupole was adjusted such that ions from *m/z* 50–2000 were efficiently transmitted. MS and MS/MS spectra were acquired alternating between low-energy and elevated-energy mode of acquisition (MS^e^).

### Protein identification using MS/MS data sets and database searching

MS/MS spectra data sets were used to generate PKL files using Protein Lynx Global Server v2.4 (Waters). Proteins were then identified using PKL files and the MASCOT search engine v2.5 (Matrix Science, London, U.K.) against the *A. hypochondriacus* transcriptome and proteome data base v1.0 (23,054 sequences) available at https://phytozome.jgi.doe.gov/ [56]. Trypsin was used as the specific protease, and one missed cleavage was allowed. The mass tolerance for precursor and fragment ions was set to 50 ppm and 0.1 Da, respectively. Carbamidomethyl cysteine was set as fixed modification and oxidation of methionine was specified as variable modification. The protein identification criteria included at least two MS/MS spectra matched at 99% level of confidence, and identifications were considered successful when significant MASCOT individual ion scores > 33 were detected, indicating identity or extensive homology statistically significant at *p* < 0.01. Identifications were considered true only for peptide matches above identity threshold FDR ≤ 5%. To estimate the relative abundance of each protein per band, it was used the exponentially modified protein abundance index (emPAI) [57]. BLAST algorithm was used for homology search against the *Viridiplantae* and *Arabidopsis thaliana* subsets of the UniProtKB database (https://www.uniprot.org/blast/).

### Bioinformatic analysis

WebLogo’s were constructed using 73 sequences of 11S globulins including Amaranthaceae, Brassicaceae, Chenopodiaceae, Cucurbitaceae, Fabace, Pedaliaceae, Poaceae and Polygonaceae families, downloaded from the viridiplantae subset of the NCBI protein sequence repository (http://weblogo.berkeley.edu/, [58]; https://www.ncbi.nlm.nih.gov/protein/, [59]). Search for conserved domains was done in different servers and databases, SMART (http://smart.embl.de, [60]), PROSITE (http://prosite.expasy.org/, [61]), Pfam (http://pfam.xfam.org, [62]), InterPro (http://www.ebi.ac.uk/interpro/, [63]) and the NCBI’s CDD (http://www.ncbi.nlm.nih.gov/entrez/query.fcgi?db=cdd, [64]). Protein domains architecture images were generated with the PROSITE MyDomains-Image Creator tool (https://prosite.expasy.org/mydomains/, [65]). Multiple sequence alignments were performed using Clustal Omega with default settings (https://www.ebi.ac.uk/Tools/msa/clustalo/, [66]). Phylogenetic analysis and percentage amino acid composition were estimated with MEGA software v7.0.21 [67], the phylogenetic tree was constructed with the neighbour-joining method and a bootstrap test of 1000 replicates and edited with iTOL [68]. For structural modelling, protein sequences were submitted to the I-TASSER server (https://zhanglab.ccmb.med.umich.edu/I-TASSER/, [69]), PDB files visualization and molecular graphics were performed with the UCSF Chimera package v1.11.2 [70].

## Additional file

Additional file 1: **Figure S1.** Triplicates of the 1D-SDS-PAGE of amaranth seed hydrophilic proteins. Each gel was obtained from an independent protein extraction. Lines: M, molecular weight marker; A, *A. hybridus*; B, *A. powellii*; C, *A. cruentus* cv Amaranteca; D, *A. hypochondriacus* cv Opaca (waxy); E, *A. hypochondriacus* cv Cristalina (non-waxy); F, *A. hypochondriacus* cv Nutrisol. Arrows at the right side indicate the differentially accumulated protein bands selected for nLC-MS/MS identification. **Figure S2.** Triplicates of the 1D-SDS-PAGE of amaranth seed hydrophobic proteins. Each gel was obtained from an independent protein extraction. Lines: M, molecular weight marker; A, *A. hybridus*; B, *A. powellii*; C, *A. cruentus* cv Amaranteca; D, *A. hypochondriacus* cv Opaca (waxy); E, *A. hypochondriacus* cv Cristalina (non-waxy); F, *A. hypochondriacus* cv Nutrisol. Arrows at the right side indicate the differentially accumulated protein bands selected for nLC-MS/MS identification. **Figure S3.** Clustal analysis of 11S globulins**.** Sequences Ah11SA (3QAC_A), Ah11SB (001411), Ah11SPheRich (006768), Ah11SHMW (021283), GmA1aB1b (1FXZ-A), GmA1bB2 (BAC55938.1), GmA2B1a (BAA00154.1), GmA3B4 (1OD5_A), GmA5A4B3 (BAD72975.1). Yellow squares: cysteine residues that form disulphide bonds between the acidic and basic subunits. Red squares: the proteolytic site for asparaginil endopeptidase that gives rise to the acid and basic subunits. Green squares: β-barrel domains. **Figure S4.** A) Representative diagram of the structural signature of the 11S globulins. The cysteines involved in the formation of the interchain disulfide bond are highly conserved. B) Cysteine contained in the acid subunit indicated in position 11. C) Cysteine contained in the basic subunit indicated in position 17. It can be observed that some amino acids are also conserved in the environment of the sequence of these cysteines, especially the site of proteolytic cleavage NG, five amino acids before the cysteine conserved in C). **Figure S5.** Amino acid composition of 11S globulins. Red squares indicate the percentage of phenylalanine. **Figure S6.** Ah11SHMW amino acid sequence. In green shows the cupin β-barrel domains of 11S globulins. The red and blue bold letters indicate the 9 repeated sequences that form the CTD-like domain and the alignment of this sequences are shown. **Table S2.** Late embryogenesis abundant proteins reported in the amaranth genome database. **Table S3.** Classification of amaranth 7S (vicilin) proteins according to the presence of specific structural domains. Proteins that were identified by LC-MS/MS in differentially accumulated bands are in bold red. (DOC 2265 kb)

Additional file 2: **Table S1.** Identification of differentially accumulated proteins amongst wild and cultivated amaranth species. Differentially accumulated bands in 1-DE (Fig. 5) were excised from gel and analysed by nLC-MS/MS. (DOCX 209 kb)

## Abbreviations

1D-SDS-PAGE: One-dimensional sodium dodecyl sulphate-polyacrylamide gel electrophoresis
AHC: Agglomerative hierarchical clustering
CDD: Conserved domain database
CHAPS: 3-[(3-Cholamidopropyl)-dimetilamonio]-propanesulfonate
ESEM: Environmental scanning electron microscope
GBSSI: Granule-bound starch synthase I
GO: Gene ontology
I-TASSER: Iterative threading assembly refinement
kDa: kilo Daltons
LEA: Late embryogenesis abundant protein
MEGA: Molecular evolutionary genetics analysis
nLC-MS/MS: Nano liquid chromatography coupled to tandem mass spectrometry
OBAP: oil body-associated protein
PCA: Principal component Açanalysis
PDB: Protein data bank
PFAM: Protein families
PMFS: phenylmethanesulfonyl fluoride
PROSITE: Protein domains, families and functional sites
RS: resistant starch
SMART: Simple modular architecture research tool
SSIII: Sucrose synthase III
SSPs: Seed storage proteins
