# Supplementary material for: Protein analysis reveals differential accumulation of late embryogenesis abundant and storage proteins in seeds of wild and cultivated amaranth species
Source: BMC Plant Biol. 2019 Feb 6;19:59. doi: 10.1186/s12870-019-1656-7 (PMC6366027; doi:10.1186/s12870-019-1656-7)
Supplement: Supplementary file 1 — Figure S1. Triplicates of the 1D-SDS-PAGE of amaranth seed hydrophilic proteins. Each gel was obtained from an independent protein extraction. Lines: M, molecular weight marker; A, A. hybridus; B, A. powellii; C, A. cruentus cv Amaranteca; D, A. hypochondriacus cv Opaca (waxy); E, A. hypochondriacus cv Cristalina (non-waxy); F, A. hypochondriacus cv Nutrisol. Arrows at the right side indicate the differentially accumulated protein bands selected for nLC-MS/MS identification. Figure S2. Triplicates of the 1D-SDS-PAGE of amaranth seed hydrophobic proteins. Each gel was obtained from an independent protein extraction. Lines: M, molecular weight marker; A, A. hybridus; B, A. powellii; C, A. cruentus cv Amaranteca; D, A. hypochondriacus cv Opaca (waxy); E, A. hypochondriacus cv Cristalina (non-waxy); F, A. hypochondriacus cv Nutrisol. Arrows at the right side indicate the differentially accumulated protein bands selected for nLC-MS/MS identification. Figure S3. Clustal analysis of 11S globulins. Sequences Ah11SA (3QAC_A), Ah11SB (001411), Ah11SPheRich (006768), Ah11SHMW (021283), GmA1aB1b (1FXZ-A), GmA1bB2 (BAC55938.1), GmA2B1a (BAA00154.1), GmA3B4 (1OD5_A), GmA5A4B3 (BAD72975.1). Yellow squares: cysteine residues that form disulphide bonds between the acidic and basic subunits. Red squares: the proteolytic site for asparaginil endopeptidase that gives rise to the acid and basic subunits. Green squares: β-barrel domains. Figure S4. A) Representative diagram of the structural signature of the 11S globulins. The cysteines involved in the formation of the interchain disulfide bond are highly conserved. B) Cysteine contained in the acid subunit indicated in position 11. C) Cysteine contained in the basic subunit indicated in position 17. It can be observed that some amino acids are also conserved in the environment of the sequence of these cysteines, especially the site of proteolytic cleavage NG, five amino acids before the cysteine conserved in C). Figure S5. Amino acid co [file 12870_2019_1656_MOESM1_ESM.doc]

**Protein analysis reveals differential accumulation of late embryogenesis abundant and storage proteins in seeds of wild and cultivated amaranth species**

Esaú Bojórquez-Velázquez^a^, Alberto Barrera-Pacheco^a^, Eduardo Espitia-Rangel^b^, Alfredo Herrera-Estrella^c^, Ana Paulina Barba de la Rosa^a,*^

^a^Instituto Potosino de Investigación Cientíﬁca y Tecnológica, A.C., San Luis Potosí, 78216, México.

^b^Instituto Nacional de Investigaciones Forestales Agrícolas y Pecuarias, Texcoco, Estado de México, 56250, México.

^c^Laboratorio Nacional de Genómica para la Biodiversidad, CINVESTAV-Irapuato, Guanajuato, 36821, México.

- Correspondence: [apbarba@ipicyt.edu.mx](mailto:apbarba@ipicyt.edu.mx), Tel +52 444 8342082

*Supplementary Material*

**Supplementary Figure S1**. Triplicates of the 1D-SDS-PAGE of amaranth seed hydrophilic proteins. Each gel was obtained from an independent protein extraction. Lanes: **M**, molecular weight marker; **A**, *A. hybridus*; **B**, *A. powellii*; **C**, *A. cruentus* cv Amaranteca; **D**, *A. hypochondriacus* cv Opaca (waxy); **E**, *A. hypochondriacus* cv Cristalina (non-waxy); **F**, *A. hypochondriacus* cv Nutrisol. Arrows at the right side indicate the differentially accumulated protein bands selected for nLC-MS/MS identification.

**Supplementary Figure S2**. Triplicates of the 1D-SDS-PAGE of amaranth seed hydrophobic proteins. Each gel was obtained from an independent protein extraction. Lanes: **M**, molecular weight marker; **A**, *A. hybridus*; **B**, *A. powellii*; **C**, *A. cruentus* cv Amaranteca; **D**, *A. hypochondriacus* cv Opaca (waxy); **E**, *A. hypochondriacus* cv Cristalina (non-waxy); **F**, *A. hypochondriacus* cv Nutrisol. Arrows at the right side indicate the differentially accumulated protein bands selected for nLC-MS/MS identification.

**Supplementary Figure S3**. Clustal analysis of 11S globulins**.** Sequences Ah11SA (3QAC_A), Ah11SB (001411), Ah11SPheRich (006768), Ah11SHMW (021283), GmA1aB1b (1FXZ-A), GmA1bB2 (BAC55938.1), GmA2B1a (BAA00154.1), GmA3B4 (1OD5_A), GmA5A4B3 (BAD72975.1). Yellow squares: cysteine residues that form disulphide bonds between the acidic and basic subunits. Red squares: the proteolytic site for asparaginil endopeptidase that gives rise to the acid and basic subunits. Green squares: β-barrel domains.

**Supplementary Figure S4**. A) Representative diagram of the structural signature of the 11S globulins. The cysteines involved in the formation of the interchain disulfide bond are highly conserved. B) Cysteine contained in the acid subunit indicated in position 11. C) Cysteine contained in the basic subunit indicated in position 17. It can be observed that some amino acids are also conserved in the environment of the sequence of these cysteines, especially the site of proteolytic cleavage NG, five amino acids before the cysteine conserved in C).

**Supplementary Figure S5**. Amino acid composition of 11S globulins. Red squares

indicate the percentage of phenylalanine.

**Supplementary Figure S6**. Ah11SHMW amino acid sequence. In green shows the cupin β-barrel domains of 11S globulins. The red and blue bold letters indicate the 9 repeated sequences that form the CTD-like domain and the alignment of this sequences are shown.

**Supplementary Table S2.** Late embryogenesis abundant proteins reported in the amaranth genome database^a^

| **Protein name** | ***A. hypochondriacus***  **proteome accession number** | **Closer orthologue**  **species/accession number** | **Pfam domain** |
| --- | --- | --- | --- |
| **Embryonic DC-8** | **AHYPO_000638-RA** | ***B. vulgaris*/XP_010683930.1** | **LEA 4** |
| **ECP63-like -X1** | **AHYPO_001171-RA** | ***C. quinoa*/XP_021737795.1** | **LEA 4** |
| D-29-like | AHYPO_004157-RA | *C. quinoa*/XP_021740545.1 | Neuromodulin N |
| DC-8 isoform X2 | AHYPO_011345-RA | *B. vulgaris*/XP_010686551.1 | Neuromodulin N |
| LEA protein | AHYPO_011345-RA | *C. quinoa*/XP_021714528.1 | - |
| LEA hydroxyproline-rich glycoprotein | AHYPO_002268-RA | *C. quinoa*/XP_021768891.1 | - |
| LEA hydroxyproline-rich glycoprotein | AHYPO_002278-RA | *C. quinoa*/XP_021774037.1 | - |
| LEA protein, 3-like | AHYPO_002961-RA | *C. quinoa*/XP_021760058.1 | - |
| LEA protein 2-like | AHYPO_002962-RA | *Q. suber*/XP_023883403.1 | - |
| LEA hydroxyproline-rich glycoprotein | AHYPO_003750-RA | *S. oleracea*/XP_021856806.1 | LEA 2 |
| LEA protein | AHYPO_004102-RA | *B. vulgaris*/XP_010690833.1 | - |
| LEA protein | AHYPO_005092-RA | *A. cruentus*/AQQ72603.1 | - |
| LEA hydroxyproline-rich glycoprotein | AHYPO_005259-RA | *C. quinoa*/XP_021760362.1 | LEA 2 |
| **LEA protein D-34** | **AHYPO_006906-RA** | ***B. vulgaris*/XP_010679058.1** | **SMP** |
| LEA protein 31 | AHYPO_006907-RA | *B. vulgaris*/XP_010679062.1 | SMP |
| LEA protein 31 | AHYPO_006909-RA | *C. quinoa*/XP_021762725.1 | SMP |
| LEA D-34-like | AHYPO_006910-RA | *C. quinoa*/XP_021764266.1 | SMP |
| LEA Lea5-like | AHYPO_007836-RA | *C. quinoa*/ XP_021763205.1 | LEA 3 |
| **Stress induced** | **AHYPO_008005-RA** | ***B.* vulgaris/XP_010676772.1** | **LEA 5** |
| LEA hydroxyproline- rich glycoprotein | AHYPO_009141-RA | *C. quinoa*/XP_021841865.1 | - |
| hydroxyproline-rich glycoprotein | AHYPO_009731-RA | *S. oleracea*/XP_021853522.1 | LEA 2 |
| LEA hydroxyproline-rich glycoprotein | AHYPO_010288-RA | *B.* vulgaris/XP_010682143.1 | LEA 2 |
| LEA D-29-like | AHYPO_010481-RA | *C. quinoa*/XP_021752787.1 | - |
| LEA protein | AHYPO_011548-RA | *C. quinoa*/XP_021767182.1 | DUF4149 |
| LEA protein 47-like | AHYPO_011838-RA | *C. quinoa*/XP_021771823.1 | SMP |
| LEA hydroxyproline-rich glycoprotein | AHYPO_012283-RA | *B. vulgaris*/XP_010689396.1 | LEA 2 |
| LEA protein group 6 | AHYPO_013245-RA | *B. vulgaris*/XP_010679579.1 | LEA 6 |
| LEA hydroxyproline-rich glycoprotein | AHYPO_013450-RA | *C. quinoa*/XP_021716626.1 | LEA 2, Why |
| LEA protein, 2 | AHYPO_013934-RA | *C. quinoa*/XP_021774726.1 | LEA 2, Why |
| LEA 47-like | AHYPO_014549-RA | *S. oleracea*/XP_021843088.1 | SMP |
| D-34-like | AHYPO_014550-RA | *S. oleracea*/XP_021841918.1 | - |
| LEA hydroxyproline-rich glycoprotein | AHYPO_016193-RA | *C. quinoa*/XP_021733033.1 | - |
| **D-34-like** | **AHYPO_016810-RA** | ***S. oleracea*/XP_021853558.1** | **SMP** |
| LEA protein | AHYPO_019517-RA | *B. vulgaris*/XP_010674693.1 | YtxH |
| **P8B6** | **AHYPO_019862-RA** | ***C. quinoa*/XP_021768105.1** | **LEA 5** |
| LEA, group 3 | AHYPO_020199-RA | *C. quinoa*/XP_021763489.1 | - |
| LEA protein 2-like | AHYPO_020201-RA | *Q. suber*/ XP_023883403.1 | - |
| LEA hydroxyproline-rich glycoprotein | AHYPO_021817-RA | *C. quinoa*/XP_021723671.1 | LEA 2 |
| **LEA** | **AHYPO_013747-RA** | ***C. quinoa*/XP_021717409.1** | **-** |

^a^Proteins that were identified by LC-MS/MS in differentially accumulated protein bands are in bold red. SMP=Seed maturation protein. Why= Water Stress and Hypersensitive response

**Supplementary Table S3**. Classification of amaranth 7S (vicilin) proteins according to the presence of specific structural domains. Proteins that were identified by LC-MS/MS in differentially accumulated bands are in bold red.

| **Protein\Domain** | **Cupin** | **Xilanase**  **Inhibitor** | **Vicilin** |
| --- | --- | --- | --- |
| **006202** |  |  | X |
| **010140(7SA)** | X |  |  |
| **006304(7SB)** | X |  |  |
| **007944(7SC)** | X |  | X |
| **018839(7SD)** | X |  | X |
| **003828** |  | X |  |
| **005737** |  | X |  |
| **007735** |  | X |  |
| **011849** |  | X |  |
| **011850** |  | X |  |
| **011853** |  | X |  |
| **011854** |  | X |  |
| **016318** |  | X |  |
