# Supplementary material for: Protein analysis reveals differential accumulation of late embryogenesis abundant and storage proteins in seeds of wild and cultivated amaranth species
Source: BMC Plant Biol. 2019 Feb 6;19:59. doi: 10.1186/s12870-019-1656-7 (PMC6366027; doi:10.1186/s12870-019-1656-7)
Supplement: Supplementary file 2 — Table S1. Identification of differentially accumulated proteins amongst wild and cultivated amaranth species. Differentially accumulated bands in 1-DE (Fig. 5) were excised from gel and analysed by nLC-MS/MS. (DOCX 209 kb) [file 12870_2019_1656_MOESM2_ESM.docx]

**Supplementary Table S1.** Amaranth proteins identified by nLC-MS/MS in differentially accumulated 1D-SDS-PAGE bands. List of peptides per protein

| **Band^a^**  **No.** | **Protein** | **Accession No.^b^** | **Ortholog ID^c^** | **Mr**  **(kDa)^d^**  **Exp.** | **Mr**  **(kDa)^e^**  **Theor.** | | **Mascot**  **Score^f^** | **PM/SC^g^** | **Identified peptides** | **Intensity pattern^h^** |
| --- | --- | --- | --- | --- | --- | --- | --- | --- | --- | --- |
| **Hydrophilic fraction** | | | | | | | | | | |
| 1 | Elongation factor 2 | 001926 | Q9ASR1 | 97.279 | 94.868 | 116 | | 8/11% | R.NMSVIAHVDHGK.S  R.IRPVLTVNK.M  R.LWGENYFDPATK.K  R.IMGPNYVPGEK.K  K.NATLTNEKETDAHPIR.A  K.EGALAEENMR.G  R.VFYASQLTAKPR.L  K.AYLPVVESFGFSGTLR.A |  |
|  | Ribonuclease TUDOR 1 | 004841 | Q8VZG7 | 97.279 | 109.142 | 58 | | 18/20% | K.AVPSGDTLVIMR.I  R.IIQGDAVPQEK.T  K.TVTLSYIMAPK.L  R.DTYDEPFAWESR.E  K.TPGASEAAIR.N  R.SLHAIVEQVR.D  R.APLTSAAVVAAASGVETAPDPFGR.E  R.MWTNYVPPVTNSK.A  K.AIHDQNFTGK.V  R.RNDVVEPYAR.E  R.QVNVSMEYSR.K + Oxidation (M)  K.DFLHFFQR.S  R.RLPAIVEYVLSGHR.F  R.LPAIVEYVLSGHR.F  R.TGTFLGSLWESK.T  K.TNMAAVLLEAGLAK.F  K.FQASFGMDR.I  K.VVVTEVLGGGK.F |  |
|  | Low-temperature-induced 65 kDa –like | 018897 | Q04980 | 97.279 | 87.076 | 51 | | 13/20% | K.YTPEQVATMDK.M  K.FGAVIEKPTGVELDPR.A  K.YVPGQEETLGWSR.T  K.SDKQFDELK.N  R.SEEHFNAAR.N  R.DSSILHQFDK.M  K.GHQWTEGSYGDR.M  R.MSNVNAIVSDK.A + Oxidation (M)  K.VADAGFTMASK.V  K.VQGSVEQKPGVGQEIGTTGVQSSR.M  R.KEDVGTGYEER.E  R.SEETSYDNAGPGLASPGK.G  R.IYEATSSWFQK.S |  |
|  | Phosphoenolpyruvate carboxylase 3 | 004468 | Q84VW9 | 97.279 | 108.392 | 46 | | 5/6% | K.RPLFGPDLPK.T  K.LADLEAAPAAVAR.L  R.ALLDEMAVVATEK.Y  R.AIPWIFAWTQTR.F  R.EILQGDPHLR.Q |  |
| 2 | Alpha-xylosidase 1 | 020003 | Q9S7Y7 | 87.977 | 93.922 | 233 | | 9/11% | R.VNWATVDSGSSR.T  R.TLTAILQVVR.N  R.WEIPNNILHR.R  K.NQYIQLSSSLPGR.K  K.DFTFDPINYPLPK.M  K.KRPFLLSR.S  R.WESVTASAR.K  R.APPDHINVHIR.E  R.EGNILALQGK.A |  |
|  | Poly [ADP-ribose] polymerase 3 | 003773 | Q9FK91 | 87.977 | 82.759 | 166 | | 12/23% | K.VSEQTEQQSPTK.K  K.AITDNLSIDQIK.E  R.NINEIAITQLITVPEQK.L  K.VGDDPSAEER.Q  R.QEEWDSVEDAIK.E  R.LFEELTGNEFLPWEK.E  K.TEALWSEFSSK.W  R.DLVAASHLIGDMSGSTLDDPLSDR.Y + Oxidation (M)  K.MIVNYLEK.T  R.AIVCSDASAEAAK.Y  K.YGFTAIDRPEGFLVLAVASLGK.E  K.EIIEVSSPPEDTESLEK.R |  |
|  | Starch branching enzyme I | 000673 | O23647 | 87.977 | 100.563 | 160 | | 16/20% | K.LTNDEGLQDLDVR.T  K.GGKEFETEEK.F  K.FIQSEDAIHDK.L  K.LDFVTSTSDHK.D  K.DKEVQTIVAK.R  R.IYEIDPLLR.N  R.NHRDHLDYR.Y  R.NEFGVWEIFLPNNADGSPAIPHGSR.V  K.IHMDTPSGIK.D  R.IYESHVGMSSTEPK.I + Oxidation (M)  R.GYHWMWDSR.L  R.LFNYGSWEVLR.Y  R.MGDIVHTLTNR.R  R.RFDLGDADYLR.Y  R.FDLGDADYLR.Y  K.IVLDSDAALFGGFNR.I |  |
|  | Elongation factor 2 | 001926 | Q9ASR1 | 87.977 | 94.868 | 132 | | 10/17% | R.NMSVIAHVDHGK.S  K.STLTDSLVAAAGIIAQEVAGDVR.M  R.IRPVLTVNK.M  R.LWGENYFDPATK.K  R.IMGPNYVPGEK.K + Oxidation (M)  K.NATLTNEKETDAHPIR.A  K.EGALAEENMR.G + Oxidation (M)  R.VFYASQLTAKPR.L  R.LLEPVYLVEIQAPEQALGGIYSVLNQK.R  K.AYLPVVESFGFSGTLR.A |  |
|  | Chaperone 1 | 008070 | P42730 | 87.977 | 100.771 | 81 | | 9/12% | R.QAISHAGGDEAADSVER.V  K.LDPVIGRDEEIR.R  K.NNPVLIGEPGVGK.T  R.IVSGDVPSNLADVR.L  R.LIALDMGALVAGAK.Y  K.YRGEFEER.L  R.YGGIQEVEAAIAR.L  K.ALAEQLFDDENLLVR.I  R.HFKPELLNR.L |  |
|  | Aminopeptidase M1 | 006828 | Q8VZH2 | 87.977 | 95.063 | 58 | | 5/7% | K.NMATTQFEPADAR.R  R.MLQSYLGADPFQR.A  K.SGSLDIVESSWIK.L  K.FFISLFEHPAGK.L  K.AKEVEEFFASR.M |  |
| 3 | Methionine synthase | 017360 | O50008 | 78.853 | 78.4925 | 529 | | 18/30% | K.FALESFWDGK.S  K.YIPSNTFAYYDQVLDTTAMLGAVPPR.Y + Oxidation (M)  K.ALGVDTVPVLVGPVSYLLLSKPAK.G  K.SFSLLSLLPK.I  K.AAGASWIQLDEPLLVR.D  K.GVTGFGFDLVR.G  K.YLFAGVVDGR.N  K.SWLAFAAQK.V  K.KLNLPILPTTTIGSFPQTVELR.R  K.KISEEEYVK.A  K.AIKEEISK.V  K.GMLTGPVTILNWSFVR.N  K.AGINVIQIDEAALR.E  R.KSEHEFYLK.W  K.SEHEFYLK.W  K.YGAGIGPGVYDIHSPR.I  R.IPPTDEIADR.I  K.YAEVQSALSNMVAAAK.Q |  |
|  | Late embryogenesis abundant protein | 013747 | Q9SKP0 | 78.853 | 72.374 | 304 | | 13/23% | K.RATQFEELASMVADSAVAAAASVDADR.K  R.ATQFEELASMVADSAVAAAASVDADR.K  R.ATQFEELASMVADSAVAAAASVDADRK.S  K.QEGANTSWETK.E  K.QGGQYHQQQDKPSAEQVSTHR.Q  K.DSLDAQESAEAR.Y  K.AKEEAANAAWEAK.E  K.EEAANAAWEAK.E  K.ASAGVGATTQYISEK.S  K.ATELAEVPVVMAR.D  K.VKENLPGGR.T  K.GGSVLGAIGETVVEIAQSTANLPSK.I  R.EMMGSMPSGGGVAGNLQK.G + 2 Oxidation (M) |  |
|  | Methionine synthase | 022179 | O50008 | 78.853 | 89.819 | 219 | | 9/16% | K.FALESFWDGK.I  K.YIPSNTFSYYDQVLDTTAMLGAVPPR.Y  K.ALGIETVPVLVGPVSYLLSSKPAK.G  K.AAGASWIQFDEPK.L  K.YLFAGVVDGR.N  K.SWLAFAAQK.I  K.GMLTGPVTILNWSFVR.N  K.YGAGIGPGVYDIHSPR.I  R.IPSTEEIVDR.V |  |
|  | Sucrose synthase | 021141 | Q9LXL5 | 78.853 | 69.915 | 124 | | 8/17% | K.QIQQQGLDITPR.I  R.FDVWPYLNTFAK.D  K.ELQTKPNLIIGNYSDGNIVATLLAQK.L  K.LDVTLGTIAHALEK.T  K.YVNSDLHWK.N  R.VVDGINVLDPK.F  R.YKPIIFSMAR.L  R.ELVNLVVVAGDR.R |  |
|  | Alpha-xylosidase 1 | 010666 | Q9S7Y7 | 78.853 | 100.260 | 111 | | 7/9% | R.DLNLYGSHPFYMDVR.S  K.ELQSVVDGYAK.M  K.DFTLDPVNFPR.K  R.GIQANVFIKR.D  R.TSNERPFILSR.S  K.YTAHWTGDNAAR.W  K.NGVTISSSVAHR.G |  |
|  | Disulfide isomerase | 015532 | Q9SRG3 | 78.853 | 56.011 | 51 | | 5/14% | R.EADGIVEYLKK.Q  K.SAEDAAAVIGDQK.V  K.SEPIPESNDEPVK.V  K.VVVADTLQEIVFNSGK.N  K.LAPILDEVAVSFENDKDIVIAK.L |  |
| 4 | Vicilin-like | 018839 | Q9SPL4^i^ | 72.738 | 61.205 | 191 | | 8/18% | R.GHVMVIPAGVTAYLVNR.G  K.LVIVK.L  K.LLNPVSNPSGK.F  R.IFSQQSEGAIIR.A  R.ASEEQISALTHEK.S  R.DSGPIQLFR.K  R.EAQELAFASSAEEIER.V  R.VFETQEEEFFFPGPR.Q |  |
|  | **Embryonic protein**  **DC-8-like** | 000638 | Q9SKP0 | 72.738 | 65.203 | 61 | | 6/10% | R.YCADQIQSAR.E  K.AGEYMDYTADK.A + Oxidation (M)  K.TGEYMDYTVDK.A + Oxidation (M)  K.GLKDVTMEK.A + Oxidation (M)  K.EVVNETAEAAK.Q  K.LTMPSDTVGR.G + Oxidation (M) |  |
| 5 | 11S globulin | 021282 | P09800^j^ | 57.852 | 78.099 | 186 | | 16/32% | R.IQAEAGVNEIWDPR.E  K.EFQCAGVTVVR.T  R.TQVEPNGLFLPHYNNAPSISYVIR.G  R.VYQGHIVALPAGVSK.W  K.WFYNDGQDR.L  R.LTIVTLFDTLNNQNQLDDILR.S  R.SFFLAGNPQGR.E  R.IFSENNILSGFDR.Q  R.QLLSQAFGIEPETVSK.I  R.VEGDLGLLIPEWDR.E  R.VEGDLGLLIPEWDREESR.R  R.RPSESYRPGQGSEWDPR.Y  R.YPGHGSQRPTHGSEWDPR.Y  R.YPGHESQRPAHGSER.D  R.YPGHESQRPAHGSERDPR.Y  R.YPGHGSQRPAHGSERDPR.Y |  |
|  | Vicilin-like | 018839 | Q9SPL4^i^ | 57.852 | 61.205 | 163 | | 8/18% | R.LLEGIENYR.V  R.GHVMVIPAGVTAYLVNR.G  K.LLNPVSNPSGK.F  R.IFSQQSEGAIIR.A  R.ASEEQISALTHEK.S  R.DSGPIQLFR.K  R.GDESTTPVHYER.I  R.VFETQEEEFFFPGPR.Q |  |
|  | Catalase | 007232 | P25819 | 57.852 | 52.508 | 112 | | 6/12% | R.APGVQTPVIVR.F  R.FSTVIHER.D  R.DAMQFFDLIR.A  K.FHWRPK.C  K.ENNFKEPGER.Y  R.NIWVSWLSQADK.S |  |
|  | Glucose-6-phosphate isomerase | 013135 | P34795 | 57.852 | 57198 | 80 | | 5/12% | K.NVVPDVWQVLDK.I  R.FLANVDPIDVAR.N  K.TFTTAETMLNAR.T  R.EWISSALGPEAVAK.H  K.YLQGSSDVPAELPTK.L |  |
| 6 | Vicilin-like | 018839 | Q9SPL4^i^ | 52.810 | 61.205 | 409 | | 14/31% | K.FREQEGNVR.V  R.LLEGIENYR.V  R.GHVMVIPAGVTAYLVNR.G  K.LLNPVSNPSGK.F  R.IFSQQSEGAIIR.A  R.ASEEQISALTHEK.S  K.SSHWPFGGK.S  R.DSGPIQLFR.K  K.QSNAFGTLFETDFDDRR.L  R.GHFEMACPHVSK.S  K.TWRGDESTTPVHYER.I  R.GDESTTPVHYER.I  R.EAQELAFASSAEEIER.V  R.VFETQEEEFFFPGPR.Q |  |
|  | 11S globulin | 021282 | P09800^j^ | 52.810 | 78.099 | 356 | | 18/34% | R.IDCQIDQLSANEPNIR.I  R.IQAEAGVNEIWDPR.E  K.EFQCAGVTVVR.T  R.TQVEPNGLFLPHYNNAPSISYVIR.G  R.EFESIRDQHQK.I  R.RVYQGHIVALPAGVSK.W  R.VYQGHIVALPAGVSK.W  R.LTIVTLFDTLNNQNQLDDILR.S  R.SFFLAGNPQGR.E  R.IFSENNILSGFDR.Q  R.QLLSQAFGIEPETVSK.I  R.VEGDLGLLIPEWDR.E  R.VEGDLGLLIPEWDREESR.R  R.RPSESYRPGQGSEWDPR.Y  R.YPGHGSQRPTHGSEWDPR.Y  R.YPGHESQRPAHGSER.D  R.YPGHESQRPAHGSERDPR.Y  R.YPGHGSQRPAHGSERDPR.Y |  |
|  | Late embryogenesis abundant protein | 001171 | Q9SKP0 | 52.810 | 45.857 | 265 | | 8/24% | R.LGELKDSAADVAK.K  K.DTEGAQDVYKDTEDR.A  R.EKGYDVEDEAAER.A  K.GYDVEDEAAER.A  K.NNILGAMGNVVDAVK.S  K.LTTPSDMVEDK.R  K.TVTVTVEESPPGAAADLLK.N  K.NADQITGQSFTPNDVGR.M |  |
|  | UTP-glucose-1-phosphate uridylyltransferase | 008585 | P57751 | 52.810 | 48.704 | 176 | | 9/25% | R.SATAGLNQISEEEK.S  K.ILSHLVQNK.N  K.IFNTNNLWVNLNAIK.R  K.VLQLETAAGAAIR.F  R.FFDNAIGINVPR.A  K.NPANPTIELGPEFK.K  K.SIPSIIELDSLK.V  K.VSIIAKPGAK.V  K.VEIPDDTVFEGF.- |  |
|  | Enolase 1 | 001183 | Q9C9C4 | 52.810 | 45.110 | 156 | | 9/29% | K.AVQNVNEIIGPALVGK.L  K.LAMQEFMILPTGASSFK.E  K.MGSEVYHSLK.T  K.VVIGMDVAASEFYR.D + Oxidation (M)  K.ISGDALKDLYK.S  K.VQIVGDDLLVTNPK.R  K.VNQIGSVTESIEAVK.M  R.AGWGVMASHR.S  R.IEEELGADAVYAGANFR.V |  |
|  | ATP synthase subunit mitochondrial-like | 001716 | Q9C5A9 | 52.810 | 59.312 | 145 | | 7/15% | R.TIAMDGTEGLVR.G + Oxidation (M)  K.TDHYLPIHR.E  K.IGLFGGAGVGK.T  K.TVLIMELINNVAK.A  R.VGLTGLTVAEHFR.D  R.DAEGQDVLLFIDNIFR.F  R.FTQANSEVSALLGR.I |  |
|  | Adenosylhomocysteinase 1 | 009349 | O23255 | 52.810 | 53.892 | 119 | | 8/16% | K.VKDLSQADFGR.L  R.TEFGPSQPFK.G  R.LVGVSEETTTGVK.R  R.HSLPDGLMR.A  K.VGVVCGYGDVGK.G  R.VTIKPQTDR.F  K.TGIIVLAEGR.L  K.VAALHLGK.L\| |  |
|  | Leucine aminopeptidase 1-like | 014952 | Q944P7 | 52.810 | 63.044 | 43 | | 5/10% | R.IGLIGLGSQPPSLSAYR.T  K.IASMYSDVLSAK.I  K.GLTFDSGGYNIK.T  K.TIEVNNTDAEGR.L  K.EVFAASDATGEK.F |  |
| 7 | Vicilin-like | 018839 | Q9SPL4^i^ | 47.210 | 61.205 | 96 | | 3/5% | R.LLEGIENYR.V  R.IFSQQSEGAIIR.A  R.DSGPIQLFR.K |  |
|  | Eukaryotic initiation factor 4A-9 | 003448 | P41376 | 47.210 | 47.072 | 45 | | 3/9% | K.GLDVIQQAQSGTGK.T  R.ILAAGVHVVVGTPGR.V  R.VLITTDLLAR.G |  |
| 8 | Phosphoglycerate kinase | 006883 | P50318 | 43.280 | 87.935 | 573 | | 19/27% | R.FYKEEEK.N  K.RVFVR.V  R.VDLNVPLDDNSNITDDTR.I  R.AAIPTITYLTGYGAK.V  K.VILCSHLGRPK.G  K.YSLKPLIPR.L  R.LSELLGIEVK.M  K.MASDSIGEEVEK.L  K.LVAELPEGGVVLLENVR.F  K.LASLADVYVNDAFGTAHR.A  K.YLKPSVAGFLMQK.E + Oxidation (M)  K.ELDYLVGAVANPK.K  K.KPFAAIVGGSK.V  K.IGVIESLLAK.V  K.VGSSLVEEDK.L  K.LELANSLMEK.A + Oxidation (M)  K.GVSLLLPTDVVIANK.F  R.KLAELSGK.G  K.GVTTIIGGGDSVAAVEK.V |  |
|  | Phosphoglycerate kinase | 019107 | Q9SAJ4 | 43.280 | 42.553 | 379 | | 14/38% | K.RVFVR.V  R.AAVPTISYLTGYGAK.V  K.VILCSHLGRPK.G  K.YSLKPLIPR.L  R.LSELLGIEVK.M  K.LVAELPEGGVLLLQNVR.F  R.FYKEEEK.N  K.YLKPSVAGFLMQK.E + Oxidation (M)  K.ELDYLVGAVANPK.K  K.KPFAAIVGGSK.V  K.IGVIESLLAK.V  K.VGSSLVEEDK.L  R.KLAELSGK.G  K.GVTTIIGGGDSVAAVEK.V |  |
|  | 11S globulin | 021282 | P09800^j^ | 43.280 | 78.099 | 140 | | 14/25% | R.IQAEAGVNEIWDPR.E  K.EFQCAGVTVVR.T  R.TQVEPNGLFLPHYNNAPSISYVIR.G  R.EFESIRDQHQK.I  R.RVYQGHIVALPAGVSK.W  R.VYQGHIVALPAGVSK.W  K.WFYNDGQDR.L  R.SFFLAGNPQGR.E  R.IFSENNILSGFDR.Q  R.QLLSQAFGIEPETVSK.I  R.VEGDLGLLIPEWDR.E  R.YPGHGSQRPTHGSEWDPR.Y  R.YPGHESQRPAHGSER.D  R.YPGHESQRPAHGSERDPR.Y |  |
|  | Vicilin-like | 018839 | Q9SPL4^i^ | 43.280 | 61.205 | 133 | | 6/13% | R.LLEGIENYR.V  R.GHVMVIPAGVTAYLVNR.G + Oxidation (M)  K.LLNPVSNPSGK.F  R.IFSQQSEGAIIR.A  R.ASEEQISALTHEK.S  K.SSHWPFGGK.S |  |
|  | Actin-7 | 019031 | P53492 | 43.280 | 41.927 | 104 | | 7/19% | R.AVFPSIVGRPR.H  K.DAYVGDEAQSK.R  K.DAYVGDEAQSKR.G  R.GYMFTTSAER.E  K.NYELPDGQVITIGAER.F  K.EITALAPSSMK.I  K.GEYDESGPSIVHR.K |  |
| 9 | Glyceraldehyde-3-phosphate dehydrogenase | 011043 | P25858 | 38.411 | 31.580 | 410 | | 9/37% | K.YDTVHGQWK.H  K.VKDETTLLFGEK.A  R.NPEEIPWK.D  R.FGIVEGLMTTVHSITATQK.T  R.AASFNIIPSSTGAAK.A  R.VPTVDVSVVDLTVR.L  K.AAIKEESEGK.L  K.AGIALNDNFVK.F  R.VVDLIVHMSK.A |  |
|  | Vicilin-like | 018839 | Q9SPL4^i^ | 38.411 | 61.205 | 403 | | 10/22% | K.FREQEGNVR.V  R.LLEGIENYR.V  R.GHVMVIPAGVTAYLVNR.G  K.LLNPVSNPSGK.F  R.IFSQQSEGAIIR.A  R.ASEEQISALTHEK.S  K.SSHWPFGGK.S  R.DSGPIQLFR.K  K.QSNAFGTLFETDFDDRR.L  R.GHFEMACPHVSK.S |  |
|  | Aldose 1-epimerase-like | 015176 | Q9STT3 | 38.411 | 32.274 | 132 | | 6/24% | R.IAGAQFTYDGIHYK.L  K.NILHGGPK.G  R.IVQGNHLSVAMR.A  K.GTPYDFLKPR.T  K.GYDINYVLDVHK.T  K.TTALTPVAVVHETK.S |  |
|  | Dehydrin Rab18-like | 003168 | P30185 | 38.411 | 26.465 | 58 | | 5/27% | R.LRDEYGNPVR.Q  R.QTDEFGNPVQHK.G  R.TGMGGYDTGTHDTGLTGVGIGTGHR.T  K.HHDTGLTGTLHR.S  K.IKEILPGGGNK.N |  |
|  | 11S globulin | 021282 | P09800^j^ | 38.411 | 78.099 | 37 | | 5/10% | R.VYQGHIVALPAGVSK.W  R.SFFLAGNPQGR.E  R.IFSENNILSGFDR.Q  R.YPGHESQRPAHGSERDPR.Y  R.YPGHGSQRPAHGSERDPR.Y |  |
| 10 | Vicilin-like | 018839 | Q9SPL4^i^ | 37.420 | 61.205 | 437 | | 13/25% | R.EREEEEEEEVGGESGAPYVFDEQHFETK.F  R.EEEEEEEVGGESGAPYVFDEQHFETK.F  R.RESFNIER.G  R.GHVMVIPAGVTAYLVNR.G  K.LLNPVSNPSGK.F  K.RIFSQQSEGAIIR.A  R.IFSQQSEGAIIR.A  R.ASEEQISALTHEK.S  K.SSHWPFGGK.S  R.DSGPIQLFR.K  K.QSNAFGTLFETDFDDR.R  K.QSNAFGTLFETDFDDRR.L  R.GHFEMACPHVSK.S |  |
|  | Lactoylglutathione lyase | 011906 | O65398 | 37.420 | 30.922 | 143 | | 4/14% | R.FLHVVYR.V  K.DPTGYLFELIQR.G  K.IVSFLDPDGWK.T  K.TVLVDNEDFLK.E |  |
|  | Malate dehydrogenase | 021284 | P57106 | 37.420 | 36.131 | 124 | | 6/29% | R.VLVTGAAGQIGYALVPMIAR.G + Oxidation (M)  K.MELVDAAFPLLK.G  K.VLVVANPANTNALILK.E  K.NVIIWGNHSSTQYPDVNHSTVK.T  K.NGEWQIVQGLPIDEVSR.K  K.MDATGAELVEEK.A |  |
| 11 | Vicilin-like | 006304 | Q9SK09 | 31.210 | 62.071 | 534 | | 15/34% | K.AEDWR.R  K.MAEEWRR.H  K.GWEEEEERER.E  R.WDEEGEEPDWRPPEPYGPAGR.R  R.GKDVFLMK.E  K.VVSTEAGEMR.V  R.MVERPLHIGFITMEPK.T  K.LGFIYNDELSER.N  R.TGSSPDSVNLYDR.D  R.NGYGWSVAVDK.H  K.VNEGDVFWIPR.Y  R.AGPFEFFGFTTSAHK.N  R.TMQGPEFATAFGLTEDRYR.D + Oxidation (M)  R.YRDIIDAQR.E  R.EAVILPSPAAAEPDERPASTKK.E |  |
|  | **Late embryogenesis abundant protein**  **(SMP)** | 006906 | Q9LJ97 | 31.210 | 28.697 | 190 | | 6/33% | R.IITESIGDQVVAR.Y  K.IVGLPAAIDRDAVTIGEALEASALSAGDKPITR.T  R.DAVTIGEALEASALSAGDKPITR.T  K.TPPGGLGAEAQSAALR.N  K.TTLGDILMDATQR.L  K.LTTNLGGVGESVAAAAR.M |  |
|  | Late embryogenesis abundant protein | 016810 | Q9LJ97 | 31.210 | 22.642 | 167 | | 5/34% | K.YGDVFPVVGELASK.A  K.GGPAATMQSAATANQR.A  R.AGLVGHLDASDVAK.H  K.VTIGEALEAVAVLAGDK.A  K.AVEQSDAAAIQAAEVR.A |  |
|  | 11S globulin | 001411 | P15456 | 31.210 | 55.753 | 130 | | 4/10% | R.LTALEPTNR.I  R.FYLAGKPQQEHSGEHQFSR.E  R.VQEGLHVIKPPSR.A  K.ADVYTPEAGR.L |  |
|  | 11S globulin | 021282 | P09800^j^ | 31.210 | 78.099 | 108 | | 7/16% | R.IQAEAGVNEIWDPR.E  R.TQVEPNGLFLPHYNNAPSISYVIR.G  K.WFYNDGQDR.L  R.LTIVTLFDTLNNQNQLDDILR.S  R.IFSENNILSGFDR.Q  R.QLLSQAFGIEPETVSK.I  R.VEGDLGLLIPEWDREESR.R |  |
|  | 60S ribosomal protein L6-3 | 005418 | Q9C9C5 | 31.210 | 25.558 | 94 | | 5/29% | K.AVDAAPVEKPAK.F  R.ASITPGTVLILLAGR.F  K.QLSSGLLLITGPFK.I  R.VNQAYVIATSTK.V  K.VDVAGVDVSNIHDK.F |  |
|  | Agglutinin | 007409 | Q38719^k^ | 31.210 | 30.397 | 80 | | 4/18% | K.ILDPLAQFEVEPSK.T  K.DVFHVIDWK.S  R.LSTDNWILVDGNDPR.E  R.ETNEAAALFR.S |  |
| 12 | Oil body-associated protein 1A | 009953 | Q9ZVY7 | 29.194 | 26.785 | 255 | | 11/35% | K.TSTTMLETATAAVQK.F  K.RLWHSHEYEVK.S  R.LWHSHEYEVK.S  K.SGVLFLPGVPGAMQR.Q  K.TIHFWQVDR.G  R.DGQLYSHLSSDVQK.R  R.DGQLYSHLSSDVQKR.Y  K.RYNVNFEK.E  R.YNVNFEK.E  R.YNVNFEKEK.E  K.GPEHGIHPLANAEGK.G |  |
|  | Vicilin-like | 006304 | Q9SK09 | 29.194 | 62.071 | 186 | | 3/9% | K.LGFIYNDELSER.N  R.TGSSPDSVNLYDRDPSFR.N  R.EAVILPSPAAAEPDERPASTK.K |  |
|  | 60S ribosomal protein L7-4 | 008528 | Q9LHP1 | 29.194 | 28.347 | 180 | | 5/18% | K.VIVPESVLK.K  K.VIVPESVLKK.Q  K.GGFYVEPEAK.L  R.VEPYVTYGYPNLK.S  R.IALTDNSIIEK.T |  |
|  | Elongation factor 1-beta 1 | 002577 | Q84WM9 | 29.194 | 24.873 | 170 | | 3/20% | K.VYSAVLEKPAETFSNASR.W  R.FSQGEAAAAPAVEEK.K  R.SVQMEGLTWGASK.L + Oxidation (M) |  |
|  | Oil body-associated protein 2A | 004342 | Q941A4 | 29.194 | 25.757 | 98 | | 7/29% | R.LIGVEYIISDR.L  R.LFEALPPDEQK.L  K.LWHSHYFEIK.S  R.VPEAVAKPELEK.F  K.FWCTWQTDR.G  K.GFAIDIITSEMK.K + Oxidation (M)  K.GFAIDIITSEMKK.I + Oxidation (M) |  |
|  | Protein synthesis inhibitor PD-S2-like | 011528 | P34967^l^ | 29.194 | 30.516 | 94 | | 4/19% | K.SATSQSYSTLLDSFR.D  K.LTGTYGFQNDLR.V  R.YYSSITGNYNDLGDR.A  R.ASVGLGAKPLNK.F |  |
| 13 | Protein synthesis inhibitor PD-S2-like | 011528 | P34967^l^ | 27.811 | 30.516 | 362 | | 13/42% | K.LTGTYGFQNDLR.V  R.VVAAPTKPAK.Y  K.ADNGIITAAFDK.N  R.LFKDAPTDIK.L  K.LIFPDVTNK.N  R.YYSSITGNYNDLGDR.A  K.FINEEIYDK.K  K.FINEEIYDKK.K  K.FDITTDK.K  K.FDITTDKK.L  K.LALMVIQTIAEATR.F  K.FGDNSGFK.C  K.SLENNWEK.T |  |
|  | Oil body-associated protein 2A | 004342 | Q941A4 | 27.811 | 25.757 | 193 | | 6/25% | R.QIETHHFVHR.L  R.LIGVEYIISDR.L  R.LFEALPPDEQK.L  R.VPEAVAKPELEK.F  K.GFAIDIITSEMK.K  K.GFAIDIITSEMKK.I + Oxidation (M) |  |
| 14 | Cysteine proteinase inhibitor 6 | 021786 | Q8H0X6 | 26.867 | 27.776 | 270 | | 11/52% | R.ESQGAANDAEIESLAR.F  R.FAVDEHNK.K  K.KENALLEFAR.V  K.EQVVAGTLHHFTIEAIDAGK.K  K.EQVVAGTLHHFTIEAIDAGKK.K  K.VWVKPWMNFK.E  K.EVPVHDPEVQNAAEHAVK.T  R.SNSLFPYELQEIAHAK.A  K.DEIFNVEVHK.S  K.SSDGNYNLNK.M  K.MGNIQPEIENQ.- + Oxidation (M) |  |
|  | Vicilin-like | 018839 | Q9SPL4^i^ | 26.867 | 61.205 | 76 | | 5/11% | R.LLEGIENYR.V  R.GHVMVIPAGVTAYLVNR.G + Oxidation (M)  K.LLNPVSNPSGK.F  R.IFSQQSEGAIIR.A  R.ASEEQISALTHEK.S |  |
|  | 11S globulin | 021282 | P09800^j^ | 26.867 | 78.099 | 46 | | 5/10% | R.SFFLAGNPQGR.E  R.IFSENNILSGFDR.Q  R.QLLSQAFGIEPETVSK.I  R.VEGDLGLLIPEWDREESR.R  R.YPGHGSQRPAHGSERDPR.Y |  |
| 15 | 11S globulin | 001411 | P15456 | 22.976 | 55.753 | 377 | | 8/19% | R.LTALEPTNR.I  R.LAVNVDDPSK.A  K.ADVYTPEAGR.L  R.GQLVVVPQNFAIVK.Q  K.QAFEDGFEWVSFK.T  K.TSENAMFQSLAGR.T  R.SLPIDVVSNIYQISR.E  K.FNRPETTLFR.S |  |
| 16 | 11S globulin | 021282 | P09800^j^ | 21.732 | 78.099 | 290 | | 10/16% | R.ITENIDDPEK.A  R.ITENIDDPEKADVFNPQGGR.L  K.ADVFNPQGGR.L  R.LTSLNSQK.L  K.LPILNYLQLSAEK.V  K.VNLYQNAIMAPNWK.I  K.INAHSIIYFTK.G  R.VQIAGHEGR.L  K.AGQEGLEWVAFLTSDEAMISPLAGR.I + Oxidation (M)  R.GLPEQVVMNSYGLSR.E |  |
|  | 11S globulin | 001411 | P15456 | 21.732 | 55.753 | 242 | | 5/12% | R.LAVNVDDPSK.A  K.ADVYTPEAGR.L  R.GQLVVVPQNFAIVK.Q  K.TSENAMFQSLAGR.T  R.SLPIDVVSNIYQISR.E |  |
| 17 | 17.6 kDa class I heat shock protein 3 | 013876 | P13853 | 17.934 | 17.918 | 182 | | 7/46% | R.SDTFSETSAFANAR.M  K.ETPEAHIFK.A  K.VEVEDGNVLR.I  K.NDTWHRVER.S  R.QFRLPENAK.V  K.AAMENGVLTVTVPK.M  K.MEAPKPEVK.A |  |
|  | Oleosin 5 | 013707 | Q9SS98 | 17.934 | 20.731 | 72 | | 5/28% | R.QAGHTVPDDLDYAK.R  R.RVADMAAYAGQK.T  R.VADMAAYAGQK.T  K.TKDVGQTIESK.A  R.ESGTHPYGGTHSTTTTGVTAR.A |  |
|  | Oleosin 5 | 015343 | Q9SS98 | 17.934 | 20.460 | 69 | | 4/21% | R.VHVHTTTTYPSYVGPK.S  R.QAGHNVPDDLDYAK.R  R.RVADMAAYAGQK.T  R.VADMAAYAGQK.T |  |
| 18 | Cyclophilin | 002428 | Q38900 | 17.428 | 17.210 | 85 | | 2/13% | K.VFFDMTVGGQPAGR.I  K.FKDENFVK.K |  |
|  | 17.4 kDa class I heat shock protein 3 | 012223 | P13853 | 17.428 | 17.357 | 34 | | 6/33% | R.ETPEAHVFK.A  K.ADLPGLKK.E  K.VLQISGER.S  R.FRLPENAK.M  K.ANMEHGVLSVTVPK.V + Oxidation (M)  K.SINIT.- |  |
| 19 | **Late embryogenesis abundant protein (LEA_5)** | 008005 | Q42489 | 15.817 | 9.661 | 152 | | 5/59% | R.REQLGHEGYQELGTK.G  R.EQLGHEGYQELGTK.G  K.EQMGTEGYQQMGR.K + 2 Oxidation (M)  K.GGLTTMDQSGEER.A  K.GIEIDESKFR.T |  |
| 20 | Vicilin-like | 018839 | Q9SPL4^i^ | 14.820 | 61.205 | 84 | | 4/8% | K.TWRGDESTTPVHYER.I  R.GDESTTPVHYER.I  R.EAQELAFASSAEEIER.V  R.VFETQEEEFFFPGPR.Q |  |
| 23 | Histone H4-like | 005348 | P59259 | 11.742 | 11.402 | 62 | | 5/41% | R.DNIQGITKPAIR.R  R.ISGLIYEETR.G  K.IFLENVIR.D  K.TVTAMDVVYALK.R + Oxidation (M)  K.TVTAMDVVYALKR.Q |  |
| 24 | **Late embryogenesis abundant protein (LEA_5)** | 019862 | Q42489 | 10.726 | 8.525 | 49 | | 3/49% | K.QGETVVPGGTGGK.S  K.SLEAQQHLAEGR.S  R.STGDKGDEAGAEDVE.- |  |
| **Hydrophobic fraction** | | | | | | | | | | |
| 27 | Granule-bound starch synthase chloroplastic amyloplastic | 011500 | Q9MAQ0 | 63.246 | 62.996 | | 1112 | 27/58% | K.LTLANNALK.S  K.SNQMSTHNGLRPLMSNIDMLR.L + 3 Oxidation (M)  K.ERFHAPFIR.S  R.FHAPFIR.S  K.TGGLGDVLGGLPPALAAR.G  R.DGWDTSVTVEFQVGNR.T  R.YFHTYK.R  R.IFVDHPLFLAR.V  R.VWGITGSK.L  K.AGADYEDNQLR.F  K.VAFCIHNIVYQGR.F  R.FALADYPR.L  R.LHLPEELRPVFEFMDGYDRPIK.G  R.KINWMK.A  K.AGILQSDR.V  R.GVELDDVVR.Q  K.EALQAEVGLPVDR.N  R.NIPLIGFIGR.L  K.GSDILAEAIPR.F  K.ENVQIVVLGTGK.E  K.QIEQLEILYPEK.A  K.FNSPLAHMIVAGADFMLIPSR.F  R.FEPCGLIQLYSMR.Y + Oxidation (M)  K.EGYTGFHMGR.F  R.FSANCDMVDPADISAVETTVHR.A + Oxidation (M)  R.ALTTYNSPAMR.E + Oxidation (M)  R.EMVINCMTQDFSWKEPAR.K |  |
|  | 11S globulin | 021282 | P09800^j^ | 63.246 | 78.099 | | 287 | 14/26% | R.IQAEAGVNEIWDPR.E  K.EFQCAGVTVVR.T  R.TQVEPNGLFLPHYNNAPSISYVIR.G  R.RVYQGHIVALPAGVSK.W  R.VYQGHIVALPAGVSK.W  K.WFYNDGQDR.L  R.SFFLAGNPQGR.E  R.IFSENNILSGFDR.Q  R.QLLSQAFGIEPETVSK.I  R.VEGDLGLLIPEWDR.E  R.VEGDLGLLIPEWDREESR.R  R.RPSESYRPGQGSEWDPR.Y  R.YPGHGSQRPTHGSEWDPR.Y  R.YPGHGSQRPAHGSERDPR.Y |  |
|  | Vicilin-like | 018839 | Q9SPL4^i^ | 63.246 | 61.205 | | 231 | 10/23% | R.LLEGIENYR.V  R.GHVMVIPAGVTAYLVNR.G + Oxidation (M)  K.LLNPVSNPSGK.F  R.IFSQQSEGAIIR.A  R.ASEEQISALTHEK.S  K.SSHWPFGGK.S  R.DSGPIQLFR.K  R.GDESTTPVHYER.I  R.EAQELAFASSAEEIER.V  R.VFETQEEEFFFPGPR.Q |  |
|  | Indole-3-aceticacid-amido synthetase | 011444 | O82243 | 63.246 | 70.168 | | 65 | 4/6% | K.GSNDVFTFK.N  R.LGAVFASAFLR.S  R.IGDVLQVTGFYNK.T  K.LLEANVEER.V |  |
| 28 | Granule-bound starch synthase chloroplastic amyloplastic | 011500 | Q9MAQ0 | 56.531 | 62.996 | | 837 | 19/43% | K.TGGLGDVLGGLPPALAAR.G  R.IFVDHPLFLAR.V  R.VWGITGSK.L  K.AGADYEDNQLR.F  K.VAFCIHNIVYQGR.F  R.FALADYPR.L  R.LHLPEELRPVFEFMDGYDRPIK.G + Oxidation (M)  K.AGILQSDR.V  R.VVTVSPYYAQELISGVER.G  R.GVELDDVVR.Q  K.EALQAEVGLPVDR.N  R.NIPLIGFIGR.L  K.GSDILAEAIPR.F  K.ENVQIVVLGTGK.E  K.QIEQLEILYPEK.A  K.FNSPLAHMIVAGADFMLIPSR.F + Oxidation (M)  R.YGTVPVVASTGGLVDTVK.E  K.EGYTGFHMGR.F  R.ALTTYNSPAMR.E |  |
|  | 11S globulin | 021282 | P09800^j^ | 56.531 | 78.099 | | 475 | 17/38% | R.IDCQIDQLSANEPNIR.I  R.IQAEAGVNEIWDPR.E  R.TQVEPNGLFLPHYNNAPSISYVIR.G  K.ALLGVTNPGCPETFEYGSSEPFSSER.D  R.RVYQGHIVALPAGVSK.W  R.VYQGHIVALPAGVSK.W  K.WFYNDGQDR.L  R.SFFLAGNPQGR.E  R.IFSENNILSGFDR.Q  R.QLLSQAFGIEPETVSK.I  R.VEGDLGLLIPEWDR.E  R.VEGDLGLLIPEWDREESR.R  R.RPSESYRPGQGSEWDPR.Y  R.YPGHGSQRPTHGSEWDPR.Y  R.YPGHESQRPAHGSERDPR.Y  R.YPGHGSQRPAHGSERDPR.Y  R.YPGHGSQRPGHGSQWEPSYPGHGSERPGHQGQER.I |  |
|  | Vicilin-like | 018839 | Q9SPL4^i^ | 56.531 | 61.205 | | 38 | 3/6% | K.LLNPVSNPSGK.F  R.IFSQQSEGAIIR.A  R.DSGPIQLFR.K |  |
| 29 | 11S globulin | 021282 | P09800^j^ | 52.413 | 78.099 | | 412 | 20/43% | R.IDCQIDQLSANEPNIR.I  R.IQAEAGVNEIWDPR.E  K.EFQCAGVTVVR.T  R.TQVEPNGLFLPHYNNAPSISYVIR.G  K.ALLGVTNPGCPETFEYGSSEPFSSER.D  R.EFESIRDQHQK.I  R.RVYQGHIVALPAGVSK.W  R.VYQGHIVALPAGVSK.W  K.WFYNDGQDR.L  R.SFFLAGNPQGR.E  R.IFSENNILSGFDR.Q  R.QLLSQAFGIEPETVSK.I  K.IQGQNDDR.G  R.VEGDLGLLIPEWDR.E  R.VEGDLGLLIPEWDREESR.R  R.RPSESYRPGQGSEWDPR.Y  R.YPGHGSQRPTHGSEWDPR.Y  R.YPGHESQRPAHGSERDPR.Y  R.YPGHGSQRPAHGSERDPR.Y  R.YPGHGSQRPGHGSQWEPSYPGHGSERPGHQGQER.I |  |
|  | Granule-bound starch synthase chloroplastic amyloplastic | 011500 | Q9MAQ0 | 52.413 | 62.996 | | 122 | 8/13% | K.TGGLGDVLGGLPPALAAR.G  R.IFVDHPLFLAR.V  K.AGADYEDNQLR.F  K.EALQAEVGLPVDR.N  R.NIPLIGFIGR.L  K.GSDILAEAIPR.F  K.ENVQIVVLGTGK.E  K.QIEQLEILYPEK.A |  |
|  | Vicilin-like | 018839 | Q9SPL4^i^ | 52.413 | 61.205 | | 119 | 10/23% | R.LLEGIENYR.V  K.LLNPVSNPSGK.F  R.IFSQQSEGAIIR.A  R.ASEEQISALTHEK.S  K.SSHWPFGGK.S  R.DSGPIQLFR.K  K.QSNAFGTLFETDFDDRR.L  R.GDESTTPVHYER.I  R.EAQELAFASSAEEIER.V  R.VFETQEEEFFFPGPR.Q |  |
|  | ATP synthase subunit mitochondrial-like | 001716 | Q9C5A9 | 52.413 | 59.312 | | 116 | 9/21% | R.TIAMDGTEGLVR.G  R.GQPVLNTGSPITIPVGR.A  R.IMNVIGEPIDER.G  K.TDHYLPIHR.E  K.VVDLLAPYQR.G  K.TVLIMELINNVAK.A  R.VGLTGLTVAEHFR.D  R.FTQANSEVSALLGR.I  R.IPSAVGYQPTLATDLGGLQER.I |  |
|  | Late embryogenesis abundant protein | 001171 | Q9SKP0 | 52.413 | 45.857 | | 114 | 5/13% | R.EKGYDVEDEAAER.A  K.GYDVEDEAAER.A  K.NNILGAMGNVVDAVK.S + Oxidation (M)  K.LTTPSDMVEDK.R + Oxidation (M)  K.NADQITGQSFTPNDVGR.M |  |
|  | Elongation factor 1-alpha-like | 001308 | P0DH99 | 52.413 | 50.962 | | 66 | 7/17% | K.STTTGHLIYK.L  K.YYCTVIDAPGHR.D  K.ARYDEIVK.E  R.STNLDWYK.G  R.LPLQDVYK.I  K.IGGIGTVPVGR.V  R.VETGVLKPGMVVTFGPTGLTTEVK.S + Oxidation (M) |  |
|  | Serine hydroxymethyltransferase 4 | 009350 | O23254 | 52.413 | 59.735 | | 66 | 4/12% | R.IMGLDLPSGGHLTHGYYTAGGK.K  K.ISATSIYFESLPYK.V  R.ANAVAIGNYLMSK.G + Oxidation (M)  K.NAVFGDSSALAPGGVR.I |  |
| 30 | Vicilin-like | 018839 | Q9SPL4^i^ | 37.952 | 61.205 | | 333 | 9/23% | R.EREEEEEEEVGGESGAPYVFDEQHFETK.F  R.LLEGIENYR.V  R.GHVMVIPAGVTAYLVNR.G + Oxidation (M)  K.LLNPVSNPSGK.F  R.IFSQQSEGAIIR.A  R.ASEEQISALTHEK.S  K.SSHWPFGGK.S  R.DSGPIQLFR.K  K.QSNAFGTLFETDFDDRR.L |  |
|  | 11S globulin | 021282 | P09800^j^ | 37.952 | 78.099 | | 210 | 13/23% | R.IQAEAGVNEIWDPR.E  R.RVYQGHIVALPAGVSK.W  R.VYQGHIVALPAGVSK.W  K.WFYNDGQDR.L  R.SFFLAGNPQGR.E  R.IFSENNILSGFDR.Q  R.QLLSQAFGIEPETVSK.I  R.VEGDLGLLIPEWDR.E  R.VEGDLGLLIPEWDREESR.R  R.RPSESYRPGQGSEWDPR.Y  R.YPGHGSQRPTHGSEWDPR.Y  R.YPGHGSQRPTHGSEWDPR.Y  R.YPGHGSQRPAHGSERDPR.Y  K.LPILNYLQLSAEK.V |  |
|  | Aldose 1-epimerase-like | 015176 | Q9STT3 | 37.952 | 32.274 | | 140 | 5/21% | R.IAGAQFTYDGIHYK.L  R.IVQGNHLSVAMR.A + Oxidation (M)  K.GTPYDFLKPR.T  K.GYDINYVLDVHK.T  K.TTALTPVAVVHETK.S |  |
|  | 11-beta-hydroxysteroid dehydrogenase 1B | 004692 | P0DKC6 | 37.952 | 74.558 | | 109 | 9/18% | K.VVLITGASSGIGEHLAYEYAK.R  K.IVVMSSSASWIPLPR.E + Oxidation (M)  K.AAMSAFYETLR.V + Oxidation (M)  K.ITIVTPGVIESEFTK.G  R.DVQVNVIPVGTVEGTAK.S  R.HVTIPAWFK.V  R.LLFLTSTDNTPR.G  K.HIVDFPGAK.A  K.AMLYPPGIQSPDVK.T + Oxidation (M) |  |
|  | Glyceraldehyde-3-phosphate dehydrogenase | 013553 | P25858 | 37.952 | 31.710 | | 83 | 6/28% | K.FGIVEGLMTTVHSITATQK.T + Oxidation (M)  R.SASYNIIPSSTGAAK.A  R.VPTVDVSVVDLTVR.L  K.AGIALNDNFVK.F  K.FVSWYDNEWGYSSR.V  R.VVDLIVHMSK.T + Oxidation (M) |  |
| 31 | Vicilin-like | 006304 | Q9SK09 | 19.472 | 62.071 | | 90 | 7/17% | K.LGFIYNDELSER.N  R.TGSSPDSVNLYDRDPSFR.N  K.VNEGDVFWIPR.Y  R.YFPFCQIASR.A  R.AGPFEFFGFTTSAHK.N  R.YRDIIDAQR.E  R.EAVILPSPAAAEPDERPASTK.K |  |
|  | Oil body-associated protein 1A | 009953 | Q9ZVY7 | 19.472 | 26.785 | | 89 | 7/35% | K.TSTTMLETATAAVQK.F  R.QCLIYDGPEADAR.L  R.LWHSHEYEVK.S  K.SGVLFLPGVPGAMQR.Q  K.TIHFWQVDR.G  R.YNVNFEK.E  R.EVDCNPGAAAHAPPR.V |  |
| 32 | Vicilin-like | 006304 | Q9SK09 | 27.110 | 62.071 | | 90 | 3/5% | K.LGFIYNDELSER.N  K.VNEGDVFWIPR.Y  R.YRDIIDAQR.E |  |
|  | Protein synthesis inhibitor PD-S2-like | 011528 | P34967^l^ | 27.110 | 30.516 | | 82 | 8/30% | K.LTGTYGFQNDLR.V  R.LFKDAPTDIK.L  R.YYSSITGNYNDLGDR.A  R.ASVGLGAKPLNK.F  K.FINEEIYDKK.K  K.FDITTDKK.L  K.FGDNSGFK.C  K.SLENNWEK.T |  |
|  | Oil body-associated protein 2A | 004342 | Q941A4 | 27.110 | 25.757 | | 45 | 4/20% | R.QIETHHFVHR.L  R.LIGVEYIISDR.L  R.VPEAVAKPELEK.F  K.GFAIDIITSEMKK.I + Oxidation (M) |  |
| 33 | Vicilin-like | 018839 | Q9SPL4^i^ | 14.750 | 61.205 | | 137 | 4/9% | R.LLEGIENYR.V  R.GDESTTPVHYER.I  R.EAQELAFASSAEEIER.V  R.VFETQEEEFFFPGPR.Q |  |
|  | Vicilin-like | 006202 | F4IQK5 | 14.750 | 59.164 | | 109 | 5/7% | R.YFECQQR.C  R.FDPQEECER.R  R.FDPQEECERR.C  R.FDPLEECQQR.C  R.FDPQEQCKQR.C |  |
|  | Nucleoside diphosphate kinase 1 | 014404 | P39207 | 14.750 | 16.185 | | 45 | 3/17% | R.GLIGDIISR.F  R.KIIGATNPLASEPGTIR.G  K.IIGATNPLASEPGTIR.G |  |
|  | 11S globulin | 001411 | P15456 | 14.750 | 55.753 | | 42 | 3/6% | R.LTALEPTNR.I  R.GQLVVVPQNFAIVK.Q  K.FNRPETTLFR.S |  |
| 34 | 11S globulin | 001411 | P15456 | 10.721 | 55.753 | | 109 | 6/15% | R.LTALEPTNR.I  R.VQEGLHVIKPPSR.A  R.GQLVVVPQNFAIVK.Q  K.TSENAMFQSLAGR.T + Oxidation (M)  R.SLPIDVVSNIYQISR.E  K.FNRPETTLFR.S |  |
|  | Vicilin-like | 006202 | F4IQK5 | 10.721 | 59.164 | | 54 | 5/7% | R.YFECQQR.C  R.FDPQEECER.R  R.FDPQEECERR.C  R.FDPLEECQQR.C  R.FDPQEQCKQR.C |  |
| ^a^Band numbers according Figure 4. ^b^Accession number according to the database reported by Clouse et al., 2016. ^c^*Arabidopsis thaliana* UniProtKB orthologue ID, for proteins without *A. thaliana* orthologues, the corresponding species are indicated by superscript. ^d^Experimental molecular weight (kDa). ^e^Theoretical molecular weight (kDa). ^f^ MASCOT score, individual ion scores >33 are statistically significant (*P*<0.01), only identifications whit peptide matches above identity threshold when FDR≤5% were considered true. ^g^Peptides Matched/Sequence Coverage. ^h^Intensity pattern histograms, different top bars lowercase letters indicates statistically significant differences (*P*<0.05), bottom capital letters indicates the corresponding amaranth species, A=*A. hybridus*, B=*A. powellii*, C=*A. cruentus* cv amaranteca, D=*A. hypochondriacus* cv Opaca, E=*A. hypochondriacus* cv Cristalina, F=*A. hypochondriacus* cv Nutrisol. ^i^Orthologue from *Macadamia integrifolia*. ^j^Orthologue from *Gossypium hirsutum*. ^k^Orthologue from *Amaranthus hypochondriacus*. ^l^Orthologue from *Phytolacca dioica* | | | | | | | | | | |
